# Significantly Improved Morphology and Efficiency of Nonhalogenated Solvent‐Processed Solar Cells Derived from a Conjugated Donor–Acceptor Block Copolymer

**DOI:** 10.1002/advs.201902470

**Published:** 2020-01-09

**Authors:** Su Hong Park, Youngseo Kim, Na Yeon Kwon, Young Woong Lee, Han Young Woo, Weon‐Sik Chae, Sungnam Park, Min Ju Cho, Dong Hoon Choi

**Affiliations:** ^1^ Department of Chemistry Research Institute for Natural Sciences Korea University 145 Anam‐Ro, Sungbuk‐gu Seoul 02841 South Korea; ^2^ Daegu Center Korea Basic Science Institute 80 Daehakro, Bukgu Daegu 41566 South Korea

**Keywords:** active layers, charge transfer, conjugated block copolymers, polymer solar cells, power conversion efficiency

## Abstract

A highly crystalline conjugated donor (D)–acceptor (A) block copolymer (PBDT2T‐*b*‐N2200) that has good solubility in nonhalogenated solvents is successfully synthesized. PBDT2T‐*b*‐N2200 shows a broad complementary absorption behavior owing to a wide‐band gap donor (PBDT2T) present as a D‐block and a narrow‐band gap acceptor (N2200) present as an A‐block. Polymer solar cells (PSCs) with conjugated block copolymer (CBCP) are fabricated using a toluene solution and PSC created with an annealed film showing the highest power conversion efficiency of 6.43%, which is 2.4 times higher than that made with an annealed blend film of PBDT2T and N2200. Compared to the blend film, the PBDT2T‐*b*‐N2200 film exhibits a highly improved surface and internal morphology, as well as a faster photoluminescence decay lifetime, indicating a more efficient photoinduced electron transfer. In addition, the PBDT2T‐*b*‐N2200 film shows high crystallinity through an effective self‐assembly of each block during thermal annealing and a predominant face‐on chain orientation favorable to a vertical‐type PSC. Moreover, the CBCP‐based PSCs exhibit an excellent shelf‐life time of over 1020 h owing to their morphological stability. From these results, a D–A block copolymer system is one of the efficient strategies to improve miscibility and morphological stability in all polymer blend systems.

## Graphical abstract

Polymer solar cells (PSCs) with conjugated block copolymer are fabricated using a PBDT2T‐*b*‐N2200 solution in a nonhalogenated solvent such as toluene. PSCs created with an annealed film show the highest power conversion efficiency of 6.43% and an excellent shelf‐life time of over 1020 h owing to their morphological stability.

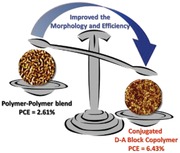

## 1 Introduction

Many types of conjugated polymers have recently been designed and synthesized to obtain a high power conversion efficiency (PCE) in all‐polymer solar cells (All‐PSCs) owing to good film formation property and high mechanical stability for flexible devices.^1^, ^2^, ^3^, ^4^, ^5^, ^6^, ^7^, ^8^, ^9^, ^10^, ^11^, ^12^, ^13^, ^14^ Through these efforts, All‐PSC has shown a high PCE of over 10%, but still has performance stability issues. A polymer–polymer blend system traditionally has difficulty controlling the miscibility, which has a significant influence on their intrinsic properties, such as the different functional groups, molecular weight, and solubility. One of the easiest solutions is the use of an appropriate solvent or additive to improve their miscibility. However, because the solubility cannot be identical in one solvent of two polymers, it is challenging to find an appropriate solvent in which both polymers are well dissolved and mixed without agglomeration. If a solvent additive is used to increase the miscibility of these components, the stability of the device cannot be ensured if a small amount of additive remains in the active layer. It can be expected that the use of polymer chains or small molecules containing well‐separated donor and acceptor moieties together as the single component active material in a PSC may overcome the disadvantages of conventional BHJ All‐PSCs bearing donor and acceptor polymers.

Over the past two decades, several research groups, including our own, have been studying new materials that covalently anchor the electron donor (D) and acceptor (A) moieties.^15^, ^16^, ^17^, ^18^, ^19^, ^20^, ^21^, ^22^, ^23^, ^24^, ^25^, ^26^, ^27^, ^28^, ^29^, ^30^, ^31^, ^32^, ^33^, ^34^, ^35^, ^36^, ^37^, ^38^, ^39^, ^40^, ^41^, ^42^, ^43^, ^44^, ^45^, ^46^, ^47^, ^48^, ^49^, ^50^, ^51^, ^52^, ^53^, ^54^, ^55^ PSCs bearing above‐mentioned materials have recently been studied in two main forms. One is a double‐cabled polymer composed mainly of a small conjugated molecular acceptor bound to the side chains of a main chain conjugated polymer donor.^20^, ^21^, ^22^, ^23^, ^24^, ^25^, ^26^, ^27^, ^28^, ^29^, ^30^, ^31^, ^32^, ^33^ The other is a conjugated block copolymer (CBCP) combining the donor block (DB) and the acceptor block (AB) in a polymer chain.^34^, ^35^, ^36^, ^37^, ^38^, ^39^, ^40^, ^41^, ^42^, ^43^, ^44^, ^45^, ^46^, ^47^, ^48^, ^49^, ^50^, ^51^, ^52^, ^53^, ^54^, ^55^, ^56^ Among them, studies on CBCP have attracted significant attention because they are relatively simple to synthesize and can lead to the design of various types of CBCPs. To date, many high‐performing CBCPs have been reported, although polythiophene (PT) derivatives are still used as a DB owing to their small polydispersity index (PDI) and single reactive end group. However, the PT‐type CBCP has many limitations in improving the PSC performance because it is laborious to tune the energy level and the absorption spectrum region in comparison to the case of other types of D–A copolymers.

In our recent study, we attempted to obtain PT‐DB free conjugated block copolymers bearing DB and AB, resulting in fairly high PCEs.^54^, ^55^, ^56^ Among them, CBCP consisting of PBDT2T and NDI‐Se can be synthesized easily based on a unique synthesis method (i.e., one‐pot polymerization). Although these types of CBCPs exhibit highly improved PCEs, their films do not show a clear difference in the morphological and crystalline behaviors compared to the case of blended films.^56^ To better understand these behaviors, we need a new molecular design of the block copolymers, which should behave distinctively from blend films.

To further improve the performance of PSCs manufactured using CBCP bearing DBs and ABs along the chain, several conditions must first be met. In particular, in terms of the CBCP structural design, the following important points should be considered. First, the DB and AB in CBCP should achieve crystallization through self‐organization among the same blocks and form a face‐on orientation, which can help with a charge carrier transport in conventional vertical PSC devices. Second, a CBCP film has a fine surface and internal morphology without a large phase separation. Third, the efficiency of the PSC can be improved if the complementary absorption range of the CBCP is broadened through a combination of DB and AB. In particular, CBCP bearing DB and AB is likely to have a high molar absorptivity within the 400–700 nm region, which shows a strong intensity in the solar spectrum. In addition, we must seriously consider the excess solvent required in the PSC manufacturing process. According to studies on highly efficient PSCs reported thus far, the most commonly used solvents and additives for preparing blend solutions for active layer deposition are all halogenated organic compounds. Therefore, in view of the mass production of PSCs, their excessive use owing to harmful effects of such halogenated compounds on humans should be suppressed.

In terms of the material design, compared to polymer donors, polymer acceptors are less developed because their energy levels are difficult to control by combining various donating and accepting moieties as monomers. Among them, poly {[*N,N*′‐bis(2‐octyldodecyl)‐naphthalene‐1,4,5,8‐bis(dicarboximide)‐2,6‐diyl]‐*alt*‐5,5′‐(2,2′‐bithiophene)} (N2200)^57^ is a promising polymer acceptor, which has many advantages such as a high crystallinity, low lying energy levels, near‐infrared absorption, and good solubility in nonhalogenated organic solvents.^10^, ^11^, ^12^, ^13^ Therefore, when designing a highly crystalline CBCP structure that exhibits good solubility in nonhalogenated solvents, N2200 was considered a very suitable acceptor unit.

In this study, we introduce a new highly crystalline conjugated D–A block copolymer (PBDT2T‐*b*‐N2200) containing a wide‐band gap benzodithiophene‐bithiophene carboxylate donor block (PBDT2T) and a narrow‐band gap N2200‐based acceptor block. PBDT2T‐*b*‐N2200 can exhibit wide complementary absorption within the 300–800 nm range. The maximum PCE of a nonhalogenated solvent‐processed CBCP‐based PSC device was measured as 6.43% after thermal annealing the active layer at 160 °C, which is remarkably higher than that of the blend film. Compared to those of a binary blend film using PBDT2T and N2200, a PBDT2T‐*b*‐N2200 film shows a dramatically improved surface and an internal morphology. This is well matched with the time‐resolved photoluminescence (TRPL) lifetime values and the distributed lifetime images. Interestingly, the annealed PBDT2T‐*b*‐N2200 films showed that DB and AB are arranged in a face‐on manner with independent/associated crystallization (i.e., crystalline–crystalline and cocrystalline structures) and easily form fine domains, achieving percolation pathways for holes and electrons. More intriguingly, the corresponding CBCP‐based PSCs exhibit a superior shelf‐life time of their photovoltaic performance of over 1020 h when stored under ambient conditions, without a significant change in morphology.

## 2 Results and Discussion

### 2.1 Polymer Synthesis and Characterization

**Figure 1:**
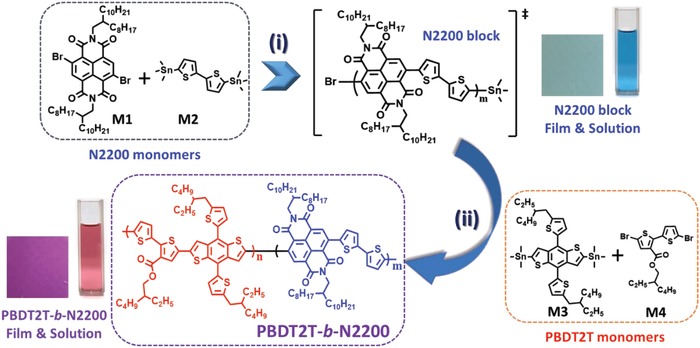
Synthesis procedure of PBDT2T‐*b*‐N2200: i) P(*o*‐tolyl)_3_, Pd_2_(dba)_3_, toluene, 110 °C, 40 min and ii) injection of M3 and M4, 110 °C, 48 h. Solution and film images of N2200 and PBDT2T‐*b*‐N2200 are included.

A highly crystalline CBCP (i.e., PBDT2T‐*b*‐N2200) bearing a wide‐band gap DB (PBDT2T) and a narrow‐band gap AB (N2200) was synthesized using a one‐pot synthesis method. The expected general structure of PBDT2T‐*b*‐N2200 is shown in **Figure** **1**.

Using a facile synthesis method reported in our previous study, we prepared the desired D–A CBCP by simply synthesizing the acceptor block first and adding only two monomers (e.g., M3 and M4) capable of forming a donor block without adding an additional catalyst in the same pot. The reaction progress in polymerization is illustrated well in Figure S1 (Supporting Information).

In our previous study, we synthesized a p‐type polymer using M3 and methyl 2,5‐dibromothiophene‐3‐carboxylate (3MT) units.^55^ In this study, bithiophene coupled with 3‐carboxylate (e.g., 2‐ethylhexyl 5,5′‐dibromo‐[2,2′‐bithiophene]‐3‐carboxylate) instead of 3MT was used in the CBCP synthesis to increase the crystallinity of p‐type domain. In addition, longer branched alkyl side chain (i.e., 2‐ethylhexyl) was introduced in the bithiophene‐3‐carboxylate monomer to obtain good solubility of the synthesized CBCP.

Contrary to our previous CBCP bearing PBDT2T and NDI‐Se, to promote the phase separation of a blend film, NDI‐Se as an AB was replaced with N2200, which has longer branched alkyl side chains and bithiophene as a donor repeating unit in a polymer backbone. In this case, the acceptor block using M1 and M2 can be used to enhance the molar absorptivity of PBDT2T‐*b*‐N2200 at 600–700 nm to improve the complementary absorption.

We determined the appropriate molecular weight of N2200 obtained through a 40 min reaction; added M3 and M4, which reacted to form a DB from the end of N2200; and carried out a Stille coupling reaction in the presence of the previously added palladium catalyst. Luckily, this study has the advantage of confirming the progress of polymerization using the four monomers by changing the color of the solution (Figure S1, Supporting Information). After 48 h, the crude product, PBDT2T‐*b*‐N2200, was precipitated in methanol and a crude polymer was purified through a Soxhlet extraction using acetone, hexane, and methylene chloride. Through the purification with this extraction, PBDT2T‐*b*‐N2200 of a relatively low molecular weight, an unreacted donor and acceptor oligomer type monomers, and a residual palladium catalyst could be removed.

To confirm that PBDT2T‐*b*‐N2200 was successfully synthesized, the number average molecular weight (*M*_n_) of the N2200 and of PBDT2T‐*b*‐N2200 was measured using GPC, as shown in Figure S2 (Supporting Information). The *M*_n_ value of the intermediate N2200 block and the final PBDT2T‐*b*‐N2200 were measured as 20.9 and 33.3 kg mol^−1^, with PDIs of 3.28 and 3.21, respectively. From these results, it was thought that the final product contained diblock and triblock copolymers. However, their exact composition could not be analyzed. To compare the various properties of PBDT2T‐*b*‐N2200 with those of the physical blend of DB and AB, PBDT2T and N2200 were also synthesized, although the MW of the two polymers did not match the MW of DB and AB present in PBDT2T‐*b*‐N2200 (Scheme S1, Supporting Information).

The solubility of the active layer material in an organic solvent is crucial for the production of PSC through a solution process. PBDT2T, N2200, and PBDT2T‐*b*‐N2200 show good solubility at room temperature (RT) in common chlorinated solvents such as chloroform, chlorobenzene, and *o*‐dichlorobenzene. Intriguingly, PBDT2T‐*b*‐N2200 is well soluble in toluene, xylenes, and mesitylene, which are nonhalogenated green solvents.

We confirmed the chemical structure of PBDT2T‐*b*‐N2200 and determined the molar ratio of the PBDT2T and N2200 units in the block copolymer by measuring ^1^H NMR an elemental analysis (EA). As shown in Figure S3 (Supporting Information), the presence of both PBDT2T and N2200 blocks in PBDT2T‐*b*‐N2200 was confirmed by integration of the peak area for the PBDT2T repeating unit (7.47–7.44, 7.31, 7.21–7.19, and 6.95 ppm for the aromatic peaks and 4.20, 2.92, and 1.74 of the aliphatic peaks) and the N2200 repeating unit (8.82, 8.51, 7.48–7.43, and 7.22–7.18 ppm for the aromatic peaks and 4.12, 3.13, and 1.98 of the aliphatic peaks).

Finally, the molar ratio of the repeating units of PBDT2T and N2200 was determined using the content of nitrogen in PBDT2T‐*b*‐N2200 obtained from repeated elemental analyses because the nitrogen element only exists in the N2200 block, as shown in Table S1 (Supporting Information). Resultantly, the molar ratios of the PBDT2T and N2200 repeating units were determined around 1:1 in PBDT2T and N2200. The PBDT2T‐*b*‐N2200 obtained after a one‐pot synthesis may contain a small amount of individual low molecular weight DB or AB owing to defective polymerization sites, but the final product has a block copolymer structure mainly containing DB and AB.^40^, ^41^, ^42^, ^43^, ^44^

### 2.2 Electrochemical Properties of PBDT2T, N2200, and PBDT2T‐*b*‐N2200

**Figure 2:**
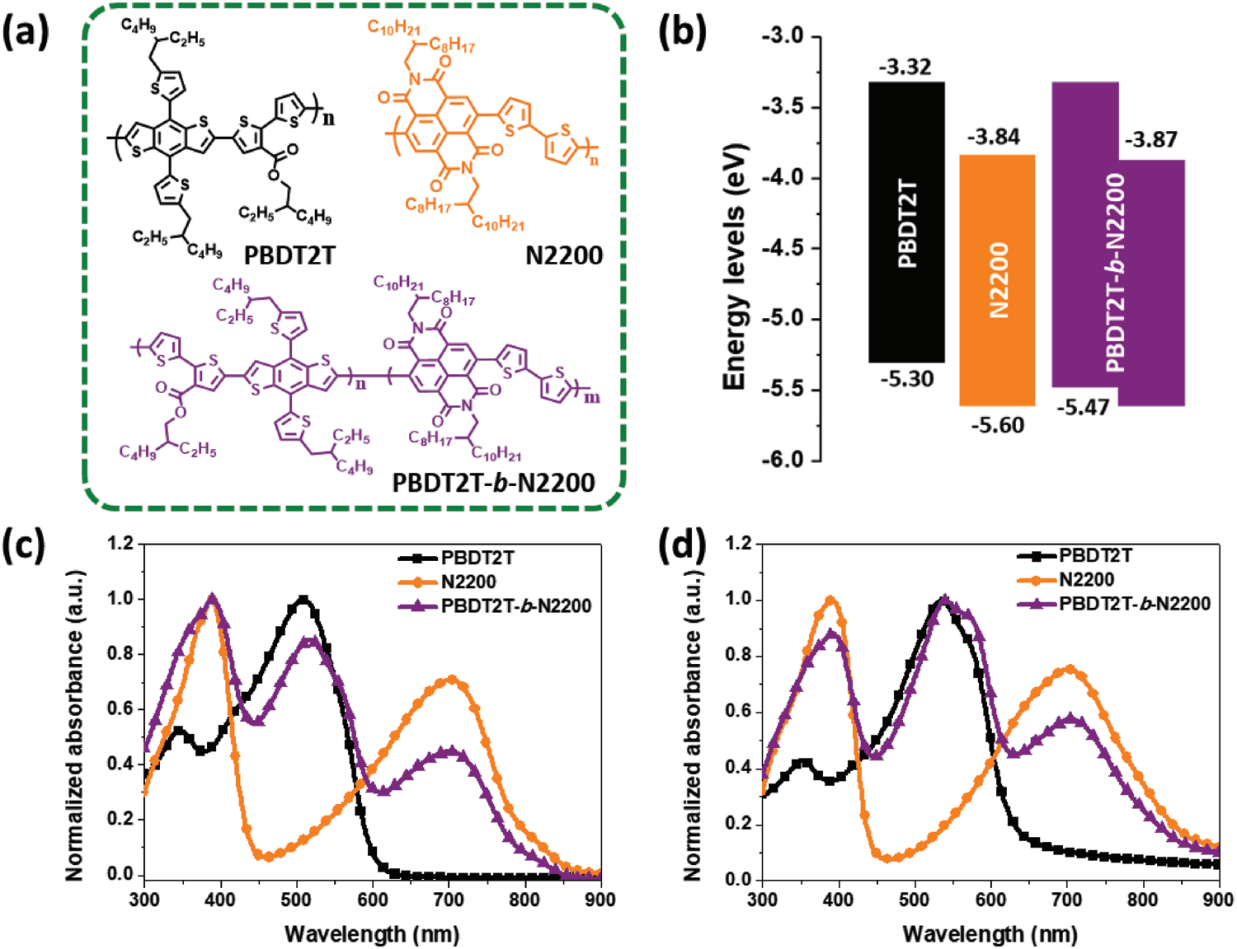
a) Polymer structures. b) Energy level diagram of the polymers. c) Normalized UV–vis absorption spectra of the polymers in chloroform and d) the thin films.

The structures of PBDT2T, N2200, and PBDT2T‐*b*‐N2200 used in this study are shown in **Figure** **2**a. The HOMO/LUMO energy levels of the PBDT2T, N2200, and PBDT2T‐*b*‐N2200 determined using a cyclic voltammetry measurement are shown in Figure S4 (Supporting Information). The oxidation/reduction potentials of PBDT2T and PBDT2T‐*b*‐N2200 were observed to be +0.86/−0.6 and +1.03 V/−0.57 V, respectively. The optical band gaps (*E*_g_) of PBDT2T and N2200 using the cutoff wavelengths of the absorption spectra in a thin film state were determined to be 1.98 and 1.47 eV, respectively. The electrochemical bandgap of PBDT2T‐*b*‐N2200 was calculated as 1.60 eV, which is between those of PBDT2T and N2200. As a result, the highest occupied molecular orbital (HOMO) and the lowest unoccupied molecular orbital (LUMO) energy levels were determined to be −5.30/−3.32, −5.60/−3.84, and −5.47/−3.87 eV for PBDT2T, N2200, and PBDT2T‐*b*‐N2200, respectively. The HOMO level of PBDT2T‐*b*‐N2200 was observed between PBDT2T and N2200 to that of PBDT2T, whereas the LUMO level is slightly lower‐lying compared to that of N2200 (Figure 2b).

### 2.3 Optical, Photoluminescence, and Time‐Resolved PL Properties of PBDT2T‐*b*‐N2200

The UV−visible absorption spectra of the synthesized PBDT2T, N2200, and PBDT2T‐*b*‐N2200 in chloroform solutions and thin films are shown in Figure 2c,d, respectively, and the corresponding data are summarized in **Table** **1**. The absorption spectrum of PBDT2T‐b‐N2200 solution at a very low concentration (≈10^−5^ m) were observed to show absorption bands of PBDT2T and N2200. The solution showed three absorption bands at 388, 517, and 700 nm, which are similar absorption features of PBDT2T and N2200. In a film state, PBDT2T‐*b*‐N2200 showed three peaks at 393, 540, and 703 nm. The as‐cast film of N2200 exhibits two distinct absorption bands, located within the ranges of 300–440 and 500–850 nm, with a maximum peak absorption observed at ≈393 and 703 nm, respectively.

**Table 1:** Physical, optical, and electrochemical properties of PBDT2T, N2200, and PBDT2T‐*b*‐N2200

	*M*_n_ [kDa]	PDI		Absorption [nm]			PL [nm]			λ_edge_ [nm]^b)^	*E*_g_^opt^ [eV]^c)^		Energy levels	
				Solution^a)^	Film^b)^		Solution^a)^	Film^b)^					HOMO [eV]^d)^	LUMO [eV]
PBDT2T	20.6	2.71		508	535		600	643		626	1.98		−5.30	−3.32^e)^
N2200	20.9	3.28		387, 703	393, 703		–	–		845	1.47		−5.60	−3.84^d)^
PBDT2T‐*b*‐N2200	33.3	3.21		388, 517, 700	393, 540, 703		596	–		837	1.48		−5.47	−3.87^d)^

^a)^ Chloroform solution

^b)^ Thin film

^c)^ The optical bandgaps were obtained from the absorption spectra cutoff of the film samples (*E*_g_^opt^ = 1240/λ_cutoff_)

^d)^ Obtained from polymer films using cyclic voltammetry and calculated according to the equation: *E*_HOMO/LUMO_ = −*e*(4.8 + *E*_ox/red_ − *E*_FC_) (eV)

^e)^ LUMO = *E_g_* (eV) + HOMO.

Comparing the absorption bands of N2200 in solution and film states, almost an identical absorption peak at 703 nm is shown, which might be due to a preorganization of N2200 molecules even in a solution. By contrast, the absorption band at 508 nm in a PBDT2T solution was shifted to 535 nm in a film state, which is ascribed to a high intermolecular interaction.

In Figure 2d, the absorption spectrum of PBDT2T‐*b*‐N2200 film showed a broad absorption range of 300–900 nm, which is a complementary absorption behavior aided by the presence of PBDT2T and N2200. In particular, the absorption at 600–800 nm owing to N2200 is quite helpful in providing a more efficient light harvesting property because of the overlapping with the solar emission spectrum with a high intensity. As with a PBDT2T film only, the absorption spectrum of PBDT2T in PBDT2T‐*b*‐N2200 was also red‐shifted in a film state, which indicates a more significant self‐association between the PBDT2T chains in a film state. From these results, no new absorption bands were observed owing to the interaction of two donor and acceptor blocks.

To investigate the molecular charge transfer (CT) phenomenon in the block copolymer, steady state and transient photoluminescence (PL) measurements were conducted using PBDT2T and PBDT2T‐*b*‐N2200 in chloroform (Figure S5a,b, Supporting Information). To suppress the intermolecular CT interaction between the polymer chains, a remarkably low concentration (≈10^−5^ m) of PBDT2T‐*b*‐N2200 in chloroform was used for the steady state and transient PL experiments. Based on the experiment results, the possible CT state formation and occurrence of PL within the visible wavelength range are depicted schematically in Figure S5c (Supporting Information). As shown in Figure S5a (Supporting Information), the PBDT2T solution emits a fluorescence peak at 600 nm but no fluorescence is observed for a N2200 in a solution.

For a very diluted solution of PBDT2T‐*b*‐N2200, it exhibits an emission peak at 596 nm. When fixing the concentration of PBDT2T in a blend solution with N2200 and PBDT2T‐*b*‐N2200, the decrement of the PL intensity in the PBDT2T‐*b*‐N2200 solution was larger than that of the blend solution. As shown in Figure S5b (Supporting Information), transient PL signals are fitted using a triexponential function, and the fitting results are summarized in Table S2 (Supporting Information). Based on our fitting results, the experiment results indicate that the average PL lifetime is shorter for PBDT2T‐*b*‐N2200 (τ_avg_ = 0.89 ns = 1/(*k*_1_ + *k*_CT_)) than for PBDT2T (τ_avg_ = 1.0 ns = 1/*k*_1_) by 110 ps.^55^, ^56^ Using a kinetic model including an intramolecular CT (*k*_CT_) from DBs to ABs, the rate constant for CT state at the local DB/AB binding region in a PBDT2T‐*b*‐N2200 structure in chloroform can be determined as *k*_CT_ = 1/τ_CT_ = 1/(8.3 ns). It has been conjectured that the photoinduced charge transfer (CT) state formation in PBDT2T‐*b*‐N2200 is more pronounced and efficient than the PBDT2T:N2200 blend solution. To clarify the reason for this different PL quenching behavior in a solution state, a transient PL measurement was accurately conducted.

### 2.4 Photovoltaic Performance of CBCP‐Based PSCs

**Figure 3:**
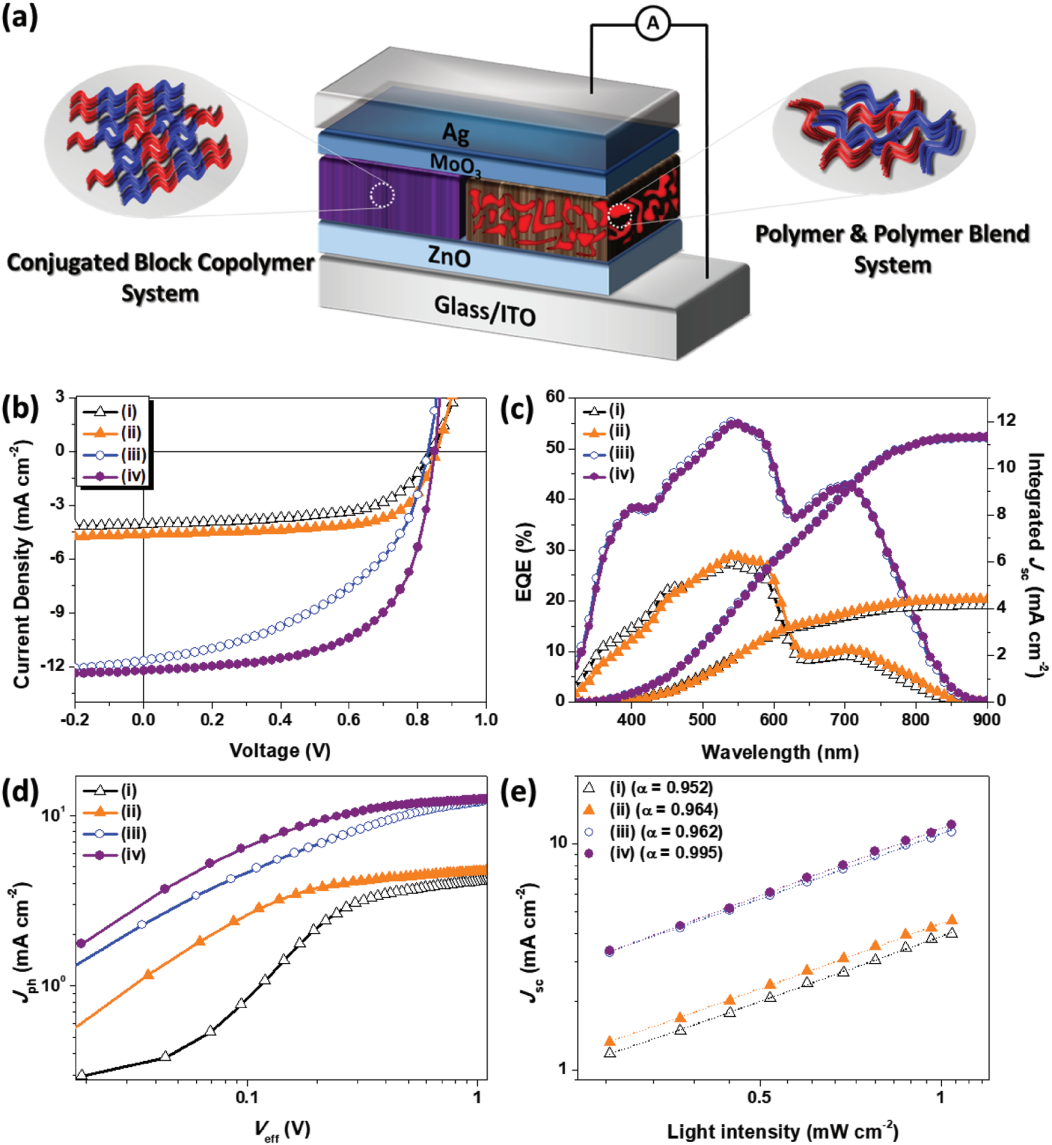
a) Inverted device structure used in this work. b) *J–V* characteristics and c) EQE spectra of PSCs with active layers of PBDT2T:N2200 blend and PBDT2T‐*b*‐N2200. d) Photocurrent density versus effective voltage characteristics. e) Plots of short‐circuit current versus light intensity. i) As‐cast blend film, ii) annealed blend film, iii) as‐cast PBDT2T‐*b*‐N2200 film, and iv) annealed PBDT2T‐*b*‐N2200 film.

To evaluate the photovoltaic performances of PSCs fabricated using a PBDT2T:N2200 blend film and a PBDT2T‐*b*‐N2200 film as the active layers, we fabricated inverted‐type PSCs with a configuration of ITO/ZnO/active layer/MoO_3_/Ag (**Figure** **3**a) The active layer materials were all dissolved in toluene, which is a nonhalogenated solvent. In addition, halogenated solvent additives (e.g., DIO and CN) have not been used at all in the fabrication of this device. The PSC in this study can be manufactured using a relatively simple process, and because the synthesized PBDT2T‐*b*‐N2200 dissolves in a nonhalogenated solvent, it should be noted that the device manufacturing was achieved through an eco‐friendly process.

The *J–V* characteristics of all PSCs fabricated in this study and characterized under an illumination of AM 1.5G simulated solar light at 100 mW cm^−2^ are shown in Figure 3 and **Table** **2**. In this study, we prepared the devices using as‐cast and annealed films with a PBDT2T:N2200 blend film and a PBDT2T‐*b*‐N2200 film as the active layers without the use of any solvent additives. The current density–voltage (*J*–*V*) curves and the corresponding EQE spectra are displayed in Figure 3b,c.

**Table 2:** Photovoltaic performance of PSCs with a device structure of ITO/ZnO/active layer/MoO_3_/Ag under AM 1.5G illumination (100 mW cm^−2^)

Active layer	*V*_oc_ [V]	*J*_sc_ [mA cm^−2^]	FF	PCE [%]	*R*_s_ [Ω cm^2^]	*R*_sh_ [kΩ cm^2^]	*µ_h_* [cm^2^ V^−1^ s^−1^]	*µ_e_* [cm^2^ V^−1^ s^−1^]	*µ*_h_/*µ*_e_
Blend	0.84(0.83 ± 0.002)	4.06(4.05 ± 0.07)	0.60(0.60 ± 0.01)	2.05(2.03 ± 0.02)	27.97	1.61	7.87 × 10^−5^	8.91 × 10^−6^	8.83
Blend^a)^	0.85(0.85 ± 0.003)	4.64(4.63 ± 0.08)	0.66(0.65 ± 0.01)	2.60(2.58 ± 0.03)	16.57	1.91	8.34 × 10^−5^	1.12 × 10^−5^	7.45
PBDT2T‐*b*‐N2200	0.83(0.83 ± 0.001)	11.63(11.41 ± 0.18)	0.47(0.46 ± 0.01)	4.54(4.41 ± 0.14)	8.98	0.36	2.54 × 10^−5^	8.78 × 10^−6^	2.89
PBDT2T‐*b*‐N2200^a)^	0.85(0.85 ± 0.002)	12.21(12.14 ± 0.15)	0.62(0.61 ± 0.01)	6.43(6.29 ± 0.13)	7.63	0.95	2.86 × 10^−5^	2.51 × 10^−5^	1.14

^a)^ Thermal annealing at 160 °C for 10 min, and average values with standard deviations were obtained from more than 20 devices.

The as‐cast PBDT2T‐*b*‐N2200 film exhibited a PCE_max_ of 4.54% with a *J*_sc_ of 11.63 mA cm^−2^, a *V*_oc_ of 0.83 V, and an FF of 0.47. When the active layer was annealed at 160 °C for 10 min, *J*_sc_ increased to 12.21 mA cm^−2^ and FF reached 0.62, resulting in 6.43% PCE. A more detailed analysis with the annealing temperature is displayed in Figure S6 and Table S3 (Supporting Information). We also found that the PSCs fabricated using the annealed PBDT2T‐*b*‐N2200 films from several devices exhibits an excellent reproducibility of PCE (Figure S7, Supporting Information). In addition, the polymers obtained from three different batches did not display significant difference, as shown in Figure S8 and Table S4 (Supporting Information). All‐PSCs made of blend films showed a relatively poor PCE of 2.0–2.6% with low *J*_sc_ values in as‐cast and annealed devices. The large difference in *J*_sc_ between PBDT2T‐*b*‐N2200‐ and PBDT2T:N2200 blend‐based PSC devices were confirmed through EQE measurements (Figure 3c; Figure S9, Supporting Information). The PSC device with an active layer fabricated from PBDT2T‐*b*‐N2200 films annealed at 160 °C exhibited a relatively higher EQE value within the 300–800 nm wavelength range (55% at 550 nm and 43% at 700 nm), compared to that of the annealed blend‐film‐based PSC device. It indicates that the PBDT2T‐*b*‐N2200 thin film exhibits enhanced light harvesting after annealing at 160 °C, resulting in relatively high *J_sc_* = 12.21 mA cm^−2^ and PCE = 6.43%. This may be attributed to the unique crystallization characteristics of the PBDT2T‐*b*‐N2200 film annealed at 160 °C, which is discussed in more detail in the grazing incidence wide‐angle X‐ray diffraction (GIWAXD) analysis of the polymer film.

The series resistance (*R*_s_) and shunt resistance (*R*_sh_) values for the four different devices are summarized in Table 2. The PSC with the annealed active layer apparently shows a decreasing tendency for *R*_s_ and an increasing tendency for *R*_sh_. In particular, the FF value of the PSC with an annealed PBDT2T‐*b*‐N2200 film is influenced greatly by both *R*_s_ (7.63 Ω cm^2^) and *R*_sh_ (950 Ω cm^2^), and a larger FF value results in improved performance of the PSC.^58^, ^59^ The device with the as‐cast film PBDT2T‐*b*‐N2200 exhibited an FF of ≈0.47, whereas the device using the film that was annealed at 160 °C for 10 min exhibited an FF of ≈0.62. This is consistent with the smaller value of *R*_sh_ (360 Ω cm^2^) of the as‐cast film shown in Table 2. Therefore, the current losses in the PSC made of an annealed CBCP film were found to be reduced after the thermal annealing process, indicating that a higher crystallization can support the prevention of the current leakage from the boundary of the electrode.

We also studied the charge generation and extraction properties of the CBCP‐based PSC devices to understand the main mechanisms related to their superior performance as compared to that of the blend‐film‐based PSC. To investigate the charge generation, dissociation, and extraction properties, we measured the dependence of the photocurrent density (*J*_ph_) on the effective voltage (*V*_eff_) of the device (Figure 3d; Figure S10, Supporting Information). This can lead us to obtain the maximum exciton generation rates (*G*_max_) of the devices characterized herein. Here, *J*_ph_ can be defined as *J*_ph_ = *J*_L_ − *J*_D_, where *J*_L_ and *J*_D_ are the current densities under illumination and in the dark, respectively. In addition, *V*_eff_ can be defined as *V*_eff_ = *V*_0_ – *V*_bias_, where *V*_0_ is the voltage at which *J*_ph_ is zero and *V*_bias_ is the applied external voltage bias.^60^, ^61^, ^62^ At high *V*_eff_ values, mobile charge carriers rapidly move toward the corresponding electrodes with a minimal recombination.

The maximum exciton generation rates (*G*_max_) can be estimated from the equation *J*_sat_ = *q*·*L*·*G*_max_, where *J*_sat_ is the saturated photocurrent density, *q* is the electronic charge, and *L* is the thickness of the active layer in the devices. The calculated *G*_max_ values of the devices are summarized in Table S5 (Supporting Information). The annealed PBDT2T‐*b*‐N2200 and blend‐film‐based device showed a saturation in the *J*_ph_ value, indicating that all photogenerated charge carriers are extracted by the electrodes. The device with an annealed PBDT2T‐*b*‐N2200 film showed the highest *G*_max_ value (8.31 × 10^27^ m^−3^ s^−1^), which was much higher than the calculated *G*_max_ values for a PBDT2T:N2200 annealed blend film device (3.1 × 10^27^ m^−3^ s^−1^). These findings indicate that the exciton generation of the PBDT2T‐*b*‐N2200 is faster than that of the PBDT2T:N2200 blend film device, which agrees with the corresponding higher *J*_sc_ values.

To investigate the extent of the carrier recombination in the active layers of the PBDT2T:N2200 blend and the PBDT2T‐*b*‐N2200 film‐based PSC, *J*_sc_ was measured as a function of the incident light intensity (*P*_light_). The data were fitted to the power‐law equation, *J*_sc_ ∝ *P*_light_ *^α^*. We also measured *J*_sc_ versus the light intensity (*P*_light_) to study the charge‐recombination behavior (Figure 3e). The relationship between *J*_sc_ and *P*_light_ can be described as *J*_sc_ ∝ (*P*_light_)*^α^*.^63^, ^64^ When all charges are collected by the electrode before a recombination, α should be equal to 1, and α < 1 indicates that a charge recombination occurs to a certain extent. After thermal annealing, the α value of the annealed PBDT2T‐*b*‐N2200 film‐based PSCs is 0.995, indicating that the molecular recombination is effectively diminished, whereas the PBDT2T:N2200 annealed blend‐film‐based device exhibits a slightly higher degree of bimolecular recombination with an α value of 0.964.

To investigate the feasibility of using nonhalogenated solvents in solution processing for CBCP‐based PSCs, we fabricated the devices using various methylbenzenes (MBs) such as toluene, *p*‐xylene, and mesitylene. In addition, we fabricated a device using CB as a control for comparison with the MB device. The detailed photovoltaic data obtained from the PSCs are shown in Figure S11 and Table S6 (Supporting Information). As a result of the device using CB, the thermal annealed PBDT2T‐*b*‐N2200 film exhibited a PCE_max_ of 5.38% with a *J*_sc_ of 11.81 mA cm^−2^, a *V*_oc_ of 0.86 V, and an FF of 0.53. Contrary to the CB solution processed PSC, the devices prepared with MB solvents displayed a slightly higher PCE exceeding 6.0%. The devices showed a similar performance after thermal annealing of the active layer. The device derived from the toluene solution showed the best performance with the highest PCE of 6.43%. This is one of the highest PCE values obtained from CBCP‐based PSCs processed using nonhalogenated solvents achieved thus far.

### 2.5 Time‐Resolved PL Spectroscopy of PBDT2T, N2200, and PBDT2T‐*b*‐N2200 Films

The photovoltaic properties of the blend films observed above are closely related to the photoinduced electron transfer phenomenon. This phenomenon can be discussed by observing the PL spectroscopy of the PBDT2T, PBDT2T:N2200 blend, and PBDT2T‐*b*‐N2200 films. PL quenching experiments were carried out to confirm the exciton dissociation and charge transfer behavior during the film states.

**Figure 4:**
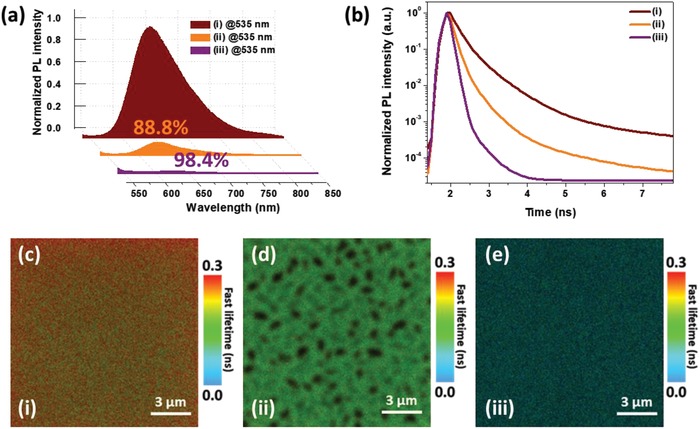
a) Photoluminescence (PL) spectra of PBDT2T, annealed blend, and annealed PBDT2T‐*b*‐N2200 in film states (excited at 535 nm). b) Time‐resolved photoluminescence (TRPL) of the PBDT2T, annealed blend, and PBDT2T‐*b*‐N2200 films. i–iii) The TRPL lifetime images of the neat PBDT2T, annealed blend, and annealed PBDT2T‐*b*‐N2200 films (the dark spots are areas of very low photon intensity). i) PBDT2T, ii) annealed blend, and iii) annealed PBDT2T‐*b*‐N2200 films.

Compared to that of the blend film, PBDT2T‐*b*‐N2200 displayed the most effective PL quenching, with an alleviation of 98.4% of the initial emission intensity, as shown in the steady state PL spectrum shown in **Figure** **4**a. This emission quenching behavior was precisely investigated through time‐resolved photoluminescence (TRPL) experiments (Figure 4b). The decay curves for the three film samples showed an initial fast decay followed by a slower decay. For the PBDT2T and PBDT2T:N2200 blend films, the variation in the PL intensity showed a triexponential decay behavior with a lifetime of 187 and 98*–*108 ps as the fast decay lifetimes, and 515 and 325–355 ps as the slow decay lifetimes, respectively. Intriguingly, in the case of a PBDT2T‐*b*‐N2200 film, a bi‐exponential PL decay was observed in which a fast decay component with a lifetime of 71 ps was followed by a slow decay component with a lifetime of 323 ps. The TRPL signal in the PBDT2T‐*b*‐N2200 film (τ_avg_ = 71 ps) decays much faster than that in the blend (τ_avg_ = 119 ps) and PBDT2T (τ_avg_ = 242 ps) films, as shown in Figure 4b. In this case, the possibly highly efficient intermolecular and intramolecular CT formation between DBs and ABs in the PBDT2T‐*b*‐N2200 film with fine morphology strongly contributes to the effective fluorescence quenching. From the lifetimes obtained, the PL quenching efficiencies were determined (see Table S7 in the Supporting Information), which are consistent with the trend qualitatively observed in the steady‐state PL spectra. In addition to the shortest lifetime found in the PBDT2T‐*b*‐N2200 film, a PL quenching efficiency of 71% is larger than that of the PBDT2T:N2200 blend film (51%).

The average exciton lifetimes correlate well with the observed quantum efficiencies, that is, the high PL quenching in the PBDT2T‐*b*‐N2200 film would cause a sufficient exciton dissociation, leading to a high EQE and *J*_sc_ of the PSCs. In addition, Figure 4c–e shows PL lifetime images of PBDT2T, PBDT2T:N2200 blend, and PBDT2T‐*b*‐N2200 films. When the blend and PBDT2T‐*b*‐N2200 films were annealed at 160 °C for 10 min, the TRPL lifetime and its uniformity in the measurement area (15 µm × 15 µm) can be observed by referring to the scale bar on the right side of the image. These images clearly reveal the uniformity of the CT domains by virtue of the color difference through the fluorescence lifetime. The blend film showed some dark spots and a nonuniform lifetime mapping on the film. These images with various colors show that PBDT2T and N2200 domains are not mixed well, and dark spots are likely to be N2200 domains that do not emit fluorescence under excitation at 535 nm. However, the lifetime distribution in a PBDT2T‐*b*‐N2200 film is extremely uniform compared to the case of the blend film, which is a good experiment result visually confirming that it has a short PL lifetime and nanosized phase formation. Therefore, the lifetime mapping techniques used in these small areas can be considered an important tool to determine the likelihood of thin films representing a photovoltaic performance (A more detailed analysis with TRPL is displayed in Figure S12 in the Supporting Information).

### 2.6 Charge‐Carrier Mobility in PBDT2T:N2200 Blend and PBDT2T‐*b*‐N2200 Films

To understand the influence of the charge transport capability on the photovoltaic performance, we measured the hole (*µ*_h_) and electron (*µ*_e_) mobilities using hole‐ and electron‐only devices, with the configurations of ITO/PEDOT:PSS/active layer/Au and ITO/ZnO/active layer/LiF/Al, respectively. The charge mobility of the active layer of the PSC was carefully evaluated using the space charge limiting current (SCLC) method. Using the modified Mott–Gurney equations, we determined the hole and electron carrier mobility by fitting the *J–V* curve in the near quadratic region.^65^ The recorded *J–V* characteristics are shown in Figure S13 (Supporting Information).

After thermal annealing, the blend and PBDT2T‐*b*‐N2200 films generally showed a higher carrier mobility than those of the as‐cast film (Table 2). The blend film based on PBDT2T and N2200 exhibited a *µ*_h_ of 8.34 × 10^−5^ cm^2^ V^−1^ s^−1^ and *µ*_e_ of 1.12 × 10^−5^ cm^2^ V^−1^ s^−1^, respectively, and the active layer based on PBDT2T‐*b*‐N2200 exhibited a *µ*_h_ of 2.86 × 10^−5^ cm^2^ V^−1^ s^−1^ and a *µ*_e_ of 2.51 × 10^−5^ cm^2^ V^−1^ s^−1^. The *µ*_h_/*µ*_e_ values of the blend film and PBDT2T‐*b*‐N2200‐based devices are 7.45 and 1.14, respectively. The balanced hole and electron mobilities play a crucial role in reducing the bimolecular recombination. Therefore, large values of *J*_sc_ and FF can be obtained. The highly balanced charge transport in the annealed film of PBDT2T‐*b*‐N2200 might be a factor increasing the FF value observed in the corresponding PSCs.

### 2.7 Morphological Characteristics of the PBDT2T:N2200 Blend Film and PBDT2T‐*b*‐N2200 Active Layer

**Figure 5:**
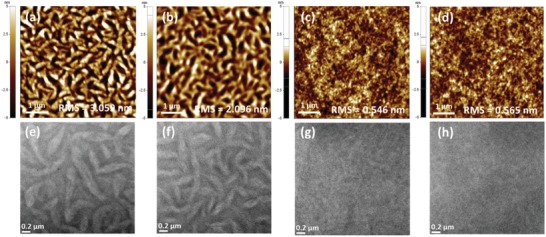
a−d) AFM height images and e−h) TEM images of the thin films fabricated from a toluene solution. a,c,e,g) As‐cast films, b,d,f,h) films annealed at 160 °C for 10 min, a,b,e,f) PBDT2T:N2200 blend films, and b,d,f,h) PBDT2T‐*b*‐N2200 films.

As shown in **Figure** **5**, the surface and internal morphology of the active layer were investigated using atomic force microscopy (AFM) and transmission electron microscopy (TEM). The as‐cast films of the PBDT2T:N2200 blend and PBDT2T‐*b*‐N2200 showed root‐mean‐square roughness (*R*_q_) values of 3.059 and 0.546 nm, respectively. After thermal annealing, the *R*_q_ values of the PBDT2T:N2200 blend and PBDT2T‐*b*‐N2200 films reached 2.096 and 0.565 nm, respectively (Figure 5a–d). As we expected, the blend film showed enlarged mesh‐ or wire‐like structures that may be attributable to their higher crystallinity of each donor or the acceptor polymer, which are not well miscible (Figure 5a,b). After thermal annealing, the phase separation behavior was not significantly improved. The TEM images of the blend film also clearly displayed a thick and fibrous texture across the entire blend film (Figure 5e,f).

Conversely, the AFM images of the PBDT2T‐*b*‐N2200 film show a relatively smooth and uniform surface and seemingly good miscibility even after thermal annealing. No microphase separation behavior could be seen in Figure 5c,d. As shown in the TEM images of Figure 5h, the internal morphology of the PBDT2T‐*b*‐N2200 film was much more homogeneous and filled with nanosized segregated crystallites; this structure is known to be more favorable for an effective exciton generation, diffusion, charge separation, and transport.

### 2.8 Grazing Incidence Wide‐Angle X‐Ray Diffraction

Grazing incidence wide‐angle X‐ray diffraction was used to study the polymer chain orientation and molecular packing of a PBDT2T:N2200 blend and PBDT2T‐*b*‐N2200 films. The diffractions of the PBDT2T and N2200 films are shown in Figure S14 and Table S8 (Supporting Information). As shown in Figure S14 (Supporting Information), the thermally annealed PBDT2T and N2200 films display distinct (100) peaks at *q_xy_* = 0.29 Å^−1^ (*d*_(100)_ = 21.34 Å) and 0.24 Å^−1^ (*d*_(100)_ = 25.81 Å) along the in‐plane direction, and (010) peaks at *q_z_* = 1.69 Å^−1^ (*d*_(010)_ = 3.70 Å) and 1.60 Å^−1^ (*d*_(010)_ = 3.92 Å) along the out‐of‐plane direction, respectively.

In Figure S14 (Supporting Information), we can trace the variation of the diffraction patterns with the annealing temperature. PBDT2T showed a high increment of diffraction intensity at *q_xy_* = 0.59 and 0.89 Å^−1^ as the annealing temperature increases. Diffraction patterns of N2200 in an in‐plane profile displayed (100), (200), and (300) diffraction peaks, and the intensity of the (300) peak increased significantly with an increase in the annealing temperature. Intriguingly, (001) and (002) diffraction peaks (i.e., *q_xy_* = 0.45 and 0.89 Å^−1^) showed a decrease in intensity with the annealing temperature, which indicates that a slight molecular rearrangement can occur to plausibly realize a face‐on orientation. The as‐cast and annealed blend film displayed most of the diffraction peaks observed, as shown in the PBDT2T and N2200 films.

**Figure 6:**
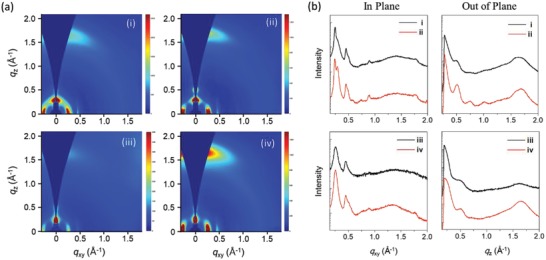
Grazing incidence wide‐angle X‐ray diffraction for PBDT2T:N2200 blend and PBDT2T‐*b*‐N2200 films: a) 2D diffraction pattern and b) in‐plane and out‐of‐plane profiles. i) As‐cast blend film, ii) annealed blend film, iii) as‐cast PBDT2T‐*b*‐N2200 film, and iv) annealed PBDT2T‐*b*‐N2200 film.

The blend film showed a very similar diffraction behavior as the PBDT2T‐*b*‐N2200 film with the annealing temperature (**Figure** **6**). The PBDT2T‐*b*‐N2200 film shows (100), (001), and (200) peaks at *q_xy_* = 0.25, 0.45, and 0.48 Å^−1^ along the in‐plane direction and one (010) peak at *q_z_* = 1.65 Å^−1^ along the out‐of‐plane direction, respectively (Figure 6; Table S9, Supporting Information). The intensity of the peak significantly increased as the annealing temperature increased. This suggests that the two polymer blocks have a predominant face‐on orientation in the PBDT2T‐*b*‐N2200 film, where the lamella plane of the polymer chain is well placed on the substrate.

To compare the π–π stacking characteristics of two polymer chains in the charge transport direction, the crystal coherence length of the (010) diffraction was revealed using GIWAXD (010) diffraction peaks and calculated using the Scherrer equation.^66^ Because the (010) peaks of the donor and acceptor polymers completely overlap, the contribution of each component to the crystallinity is difficult to distinguish. The crystal coherence length of the (010) diffraction peaks increased owing to the decrease in the full width at half‐maximum (FWHM) after thermal annealing.

Consequently, in the PBDT2T‐*b*‐N2200 film, the donor and acceptor blocks underwent double‐crystallization behavior as confirmed through the appearance of the diffraction peaks of PBDT2T and N2200 in the diffraction profiles of the PBDT2T‐*b*‐N2200 films; in addition, the face‐on crystalline orientation of the polymer chains and their aggregates is thought to be the most favorable orientation to bring about an intra/intermolecular facile exciton dissociation, efficient hole/electron generation, and charge carrier transport in the PSCs.

From these results, it can be said that the working mechanism of the CBCP‐based PSC is almost similar to that of the conventional BHJ PSC device with a common binary blend. Charge transport pathways can only be formed when crystalline–crystalline domains with the same donor or acceptor blocks are formed in the CBCP. Although co‐crystallization between donor blocks and acceptor blocks is possible, the individual crystallization between homogeneous blocks (i.e., D‐blocks or A‐blocks) can provide a very beneficial effect on device performance.^56^

### 2.9 Shelf‐Life Stability of the CBCP‐Based PSCs

We investigated the stability of the PSC device by storing it under atmospheric conditions for up to 1020 h without encapsulation. The stability of the PSC device was assessed using *J–V* measurements under ambient conditions according to the method recommended by the ISOS‐D‐1 (shelf) protocol.^67^

**Figure 7:**
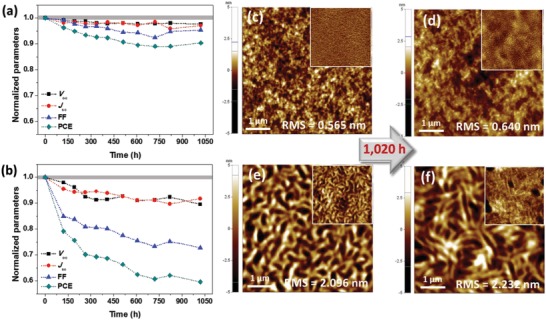
Evaluation of PSC performance stability under ambient conditions of up to 1020 h storage in a) PBDT2T‐*b*‐N2200 and b) PBDT2T:N2200 blend. AFM height images of the c,d) PBDT2T‐*b*‐N2200 film and e,f) PBDT2T:N2200 blend film. c,e) Before aging and d,f) after aging under ambient conditions in the dark at up to 1020 h. Insets show the phase images.

**Figure** **7**a,b shows the changes in the photovoltaic parameters according to the storage time for PSCs fabricated using the blend film and PBDT2T‐*b*‐N2200 active layer. The changes in the representative *V*_oc_, *J*_sc_, FF, and PCE values were determined with respect to the storage time under ambient conditions to clearly show the effect of aging on the photovoltaic parameters of the PSC during long‐term storage. After a maximum storage time of 1020 h, 90.1% of the initial PCE was maintained for the CBCP‐based PSC, whereas the blend‐film‐based devices maintained 59.5% of the initial PCE.

Furthermore, to investigate the effect of changes in the surface morphology on the shelf‐life, we used AFM to observe the topographical changes in the blend and PBDT2T‐*b*‐N2200 films under storage in ambient conditions over a long period of time, as shown in Figure 7c*–*f. The PBDT2T‐*b*‐N2200 annealed film was observed to maintain a relatively nanosized morphology even after 1020 h under ambient conditions. However, in the case of the blend film of PBDT2T and N2200, a relatively large extent of aggregation occurs, as confirmed through the AFM images (A more detailed analysis with surface profiles is displayed in Figure S15, Supporting Information). As can be seen from the morphology of the PBDT2T‐*b*‐N2200 film, an excellent shelf‐life stability was observed in the PSC when using the CBCP‐based active layer.

## 3 Conclusions

In this study, we demonstrated a novel fully conjugated D–A block copolymer (PBDT2T‐*b*‐N2200) showing high crystallinity and wide complementary absorption behavior for application to PSC devices. The large phase separation observed in the blend film of a donor polymer and an acceptor polymer was significantly improved using the block copolymer framework. The DB and AB of PBDT2T‐*b*‐N2200 showed a high crystallinity between the same blocks through a self‐assembly and improved the crystallinity through thermal annealing. Further, in the annealed PBDT2T‐*b*‐N2200 film, the polymer chains were remarkably observed in a face‐on orientation.

For the CBCP‐based PSC prepared by annealing an active layer prepared from the nonhalogenated solvent proposed in this study, the highest PCE of 6.43% was shown, which is the highest value reported thus far among all devices including the active layer produced by conjugated block copolymers. In addition, the high efficiency PSC used in this study demonstrated an excellent shelf‐life even after 1020 h of aging owing to the morphological stability of the CBCP‐based active layer.

## Conflict of Interest

The authors declare no conflict of interest.

## Supporting information

Supporting Information
