# Supplementary material for: Significantly Improved Morphology and Efficiency of Nonhalogenated Solvent‐Processed Solar Cells Derived from a Conjugated Donor–Acceptor Block Copolymer
Source: Adv Sci (Weinh). 2020 Jan 9;7(4):1902470. doi: 10.1002/advs.201902470 (PMC7029657; doi:10.1002/advs.201902470)
Supplement: Supplementary file 1 — Supporting Information [file ADVS-7-1902470-s001.pdf]

Copyright WILEY-VCH Verlag GmbH & Co. KGaA, 69469 Weinheim, Germany, 2020.

ADVANCED SCIENCE
Open Access

Supporting Information

for *Adv. Sci.,* DOI: 10.1002/advs.201902470

Significantly Improved Morphology and Efficiency of Nonhalogenated Solvent-Processed Solar Cells Derived from a Conjugated Donor–Acceptor Block Copolymer

Su Hong Park, Youngseo Kim, Na Yeon Kwon, Young Woong Lee, Han Young Woo, Weon-Sik Chae, Sungnam Park, Min Ju Cho, and Dong Hoon Choi**

Supporting Information

Significantly Improved the Morphology and Efficiency of Non-halogenated Solvent Processed Solar Cells Derived from a Conjugated Donor-Acceptor Block Copolymer

Su Hong Park [a], Youngseo Kim [a], Na Yeon Kwon [a], Young Woong Lee [a], Han Young Woo [a], Weon-Sik Chae [b], Sungnam Park [a], Min Ju Cho *,[a] and Dong Hoon Choi *,[a]

[a] Department of Chemistry, Research Institute for Natural Sciences, Korea University, 145 Anam-Ro, Sungbuk-gu, Seoul 02841 South Korea

[b] Daegu Center, Korea Basic Science Institute, 80 Daehakro, Bukgu, Daegu, Korea, 41566.

*Corresponding authors: M. J. Cho (chominju@korea.ac.kr), D. H. Choi (dhchoi8803@korea.ac.kr)

Research trends of PSCs bearing molecular dyad, double-cable polymer, and D-A block copolymer

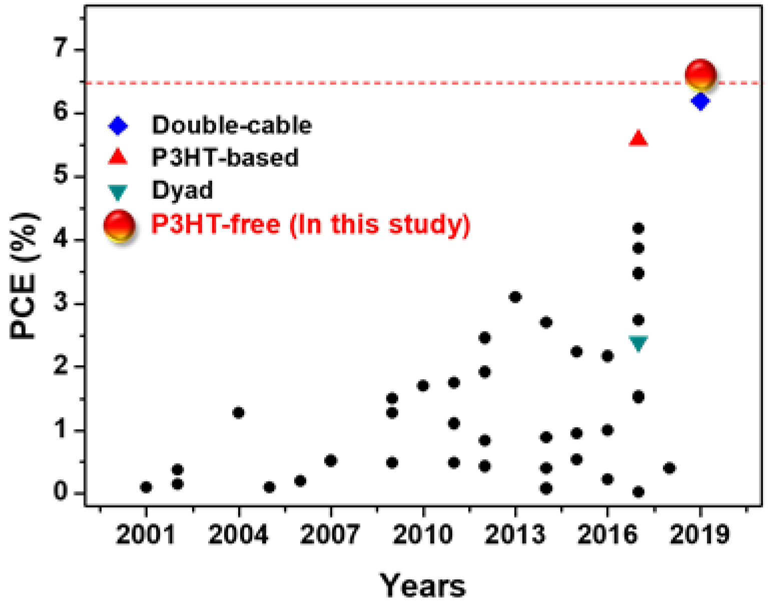

Year	Type	PCE (%)	Reference
**2017**	P3HT-based Block Copolymer	5.58	S1
**2017**	Dyad	2.44	S2
**2019**	Double-cable Polymer	6.32	S3
**2019**	P3HT-free Block Copolymer	6.43	In this study

EXPERIMENTAL SECTION

Materials

All chemicals used for the synthesis of the PBDT2T-*b*-N2200 were purchased from Sigma-Aldrich, Acros Organics Co., and Tokyo Chemical Industry and used without further purification. The reagent grade solvents used in this experiment were freshly dried using standard distillation methods.

Synthesis

Polymerization of N2200

For the N2200 polymer, M1 (205.10 mg, 20.82 mmol, 1 eq.) and M2 (102.41 mg, 20.82 mmol, 1 eq.) were dissolved in the mixture of dry toluene (8 mL) and anhydrous DMF (2 mL) by adding $Pd_2(dba)_3$ and P(*o*-tolyl)$_3$. The reaction mixture was heated and stirred for 40 min at 110 °C. Then, the solution was cooled and precipitated in methanol (300 mL). The unreacted monomer and oligomers were separated using the Soxhlet extraction method with acetone, n-hexane, methyl chloride, and chloroform sequentially. The extracted chloroform fraction was precipitated into methanol (300 mL) and dried in vacuo at 60 °C for 24 h (N2200: M_n of 20.9 kg mol^{-1}; PDI of 3.28).

Polymerization of PBDT2T

For the PBDT2T polymer, M3 (188.34 mg, 20.82 mmol, 1 eq.), M4 (100.02 mg, 20.82 mmol, 1 eq.), $Pd_2(dba)_3$, and P(*o*-tolyl)$_3$ were added to a two-neck round bottom flask. Dry toluene (10 mL) was then added and the mixture was degassed. The reactor was stirred and refluxed

for 24 h at 110 °C. After cooling to RT, the resulting solution was precipitated into methanol (300 mL). The byproducts, namely, the unreacted monomer and oligomers, were removed through a Soxhlet extraction method using acetone, n-hexane, methyl chloride, and chloroform successively. The concentrated chloroform fraction was then precipitated into methanol (300 mL) and dried under a vacuum at 60 °C for 24 h (PBDT2T: M_n of 20.6 kg mol^{-1}; PDI of 2.71).

One-pot synthesis of PBDT2T-*b*-N2200

To prepare the N2200 monomers, M1 (205.10 mg, 20.82 mmol, 1 eq.), M2 (102.41 mg, 20.82 mmol, 1 eq.), $Pd_2(dba)_3$, and P(*o*-tolyl)$_3$ were dissolved in toluene (9 mL) and anhydrous DMF (1 mL) and the reaction mixture was degassed. The temperature was gradually increased to 110 °C while stirring the reaction mixture. After 40 min, the mixed toluene solution (3 mL) of M3 (188.34 mg, 20.82 mmol, 1 eq.) and M4 (100.02 mg, 20.82 mmol, 1 eq.) was added to a reactor in which N2200 was prepared. The reaction mixture was kept under stirring at 110 °C for 48 h. The resulting solution was cooled and precipitated in methanol (300 mL). The byproducts and residual oligomers were eliminated through a Soxhlet extraction with acetone, hexane, methyl chloride, and chloroform. The chloroform fraction was then poured into methanol (300 mL) and the precipitated products were dried in vacuo at 60 °C for 24 h (PBDT2T-*b*-N2200: M_n of 33.3 kg mol^{-1}; PDI of 3.21). Elemental analyses calculated for $(C_{62}H_{88}N_2O_4S_2)$m-($C_{51}H_{60}O_2S_6$)n determined the following: C, 71.33; H, 7.83; N, 1.46; and S, 11.82.

Characterization and measurements

Instrumentation

The ^{1}H NMR spectra were recorded using a Bruker 500 MHz spectrometer (Ascend 500, Bruker) for a structural analysis of all synthesized compounds. The number average molecular weights (M_n) and PDIs of the PBDT2T, N2200, and PBDT2T-*b*-N2200 were estimated using gel permeation chromatography (GPC; Agilent GPC 1200 series) at 80 °C with *o*-dichlorobenzene (*o*-DCB) as the eluent and polystyrene (PS) as the standard. The absorption spectra of PBDT2T, N2200, and PBDT2T-*b*-N2200 in chloroform (CF) solutions and thin films were recorded using a UV−vis absorption spectrometer (Agilent 8453, photodiode array, λ = 190−1100 nm). The electrochemical properties were characterized through cyclic voltammetry (CV, eDAQ EA161) using an electrolyte solution prepared by dissolving tetrabutylammonium hexafluorophosphate (Bu_4NPF_6) in acetonitrile. A Pt wire and Ag/AgCl were used as the counter and reference electrodes, respectively.

Grazing incidence wide-angle X-ray diffraction (GIWAXD) measurements were conducted at the 9 Å beamline (energy = 11.015 keV, pixel size = 88.6 μm, λ = 1.12199 Å, 2θ = 0−20°) of the Pohang Accelerator Laboratory. Films were prepared by spin-coating the polymer solutions onto a SiO_2 wafer. The surface morphologies of the films were explored using an atomic force microscope (XE-100, Advanced Scanning Probe Microscope, PSIA) with a silicon cantilever. TEM was applied to observe the internal morphology of thin films (Tecnai G2F30 transmission electron microscope, FEI Inc.; accelerating voltage = 300 kV). The

samples used for TEM observations were prepared by coating the polymer solution on a carbon-coated copper grid.

Time-resolved photoluminescence spectroscopy (Fluorescence confocal and lifetime imaging)

A fluorescence confocal imaging and transient lifetime imaging study was conducted using an inverted-type scanning confocal microscope (SP8 FALCON, Leica Microsystems, Germany) with a 100× (oil) objective lens. The lifetime measurements were conducted at KBSI, Daegu Center, Korea. A picosecond laser line (535 nm) from a white light laser (WLL) source was used as an excitation source. A hybrid photon detector was used to collect emissions from the samples. Fluorescence confocal and lifetime images of 512 pixels × 512 pixels were simultaneously recorded using a galvo-stage and time-correlated single-photon counting technique. Exponential fittings for the obtained fluorescence decays were applied using the Leica suited software (LAS X Ver.3.5.2).

PSC device fabrication

The device was fabricated using a solution process and had an inverted device configuration of glass/ITO/ZnO/active layer/MoO_3/Ag. An ITO (150 nm)-coated glass was cleaned using ultrasonication in deionized water and isopropyl alcohol for 10 min, respectively. The dried ITO glass was subjected to UV−ozone treatment for 20 min. ZnO (40 nm) as an electron transport layer was spin-coated on the top of ITO glass using a ZnO precursor solution at 3,000 rpm for 40 s. After drying at 165 °C for 1 h, it was transferred to a glove box filled with nitrogen gas for use.

The active layer of the PSC was spin-coated on the ZnO layer in a 15 mg mL^{-1} anhydrous toluene solution. The prepared solution was then spin-coated onto the ZnO layer. The resulting active layer was 80−100 nm thick. Finally, a MoO_3 (10 nm) layer and a Ag (100 nm) layer were deposited on the active layer using a thermal evaporator to form a 4 mm^2 active region through a shadow mask. The *J*−*V* curves of the devices were measured using a Keithley 2400 source meter under simulated AM 1.5G illumination (100 mW cm^{-2}). The external quantum efficiency (EQE) spectra were recorded using a certified EQE instrument (McScience Inc., EQX 3100).

Measurement of charge mobility through space-charge-limited current (SCLC)

Hole-only devices, with a structure of ITO/PEDOT:PSS/active layer/Au, and electron-only devices with a configuration of ITO/ZnO/active layer/LiF/Al, were fabricated. The hole and electron carrier mobilities were determined using the modified Mott-Gurney equation, $J = (9/8)\varepsilon_0\varepsilon_r\mu(V^2/L^3)$, where J is the current density, μ is the mobility, and V is the applied voltage, and the device thickness, L, is defined. Moreover, ε_0 is the vacuum permittivity and ε_r is the relative permittivity. The mobility was calculated from the slope of the *J*–*V* plots.

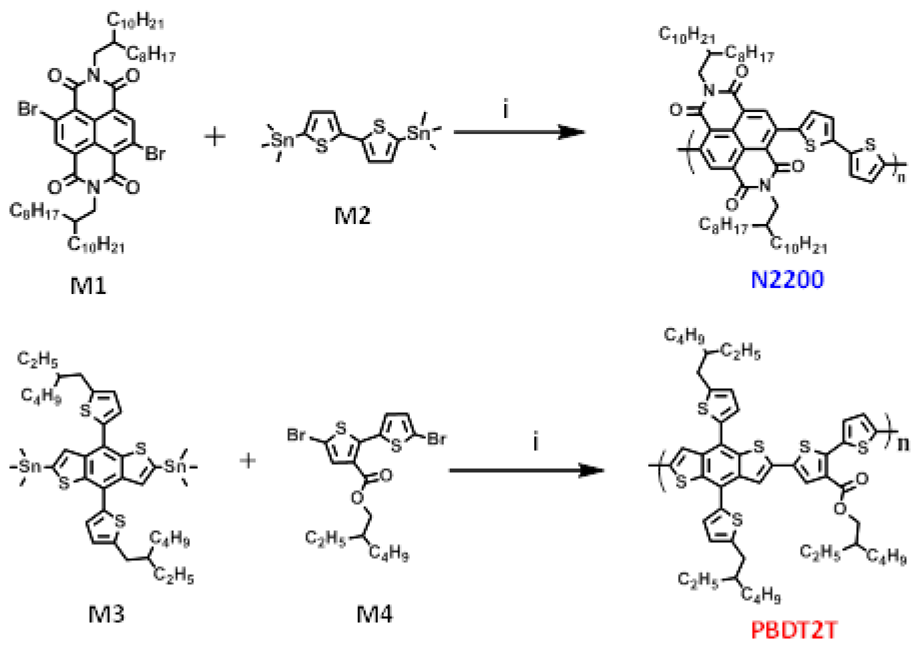

Scheme S1. Synthetic procedure of N2200 and PBDT2T: i) P(*o*-tolyl)$_3$, $Pd_2(dba)_3$, toluene.

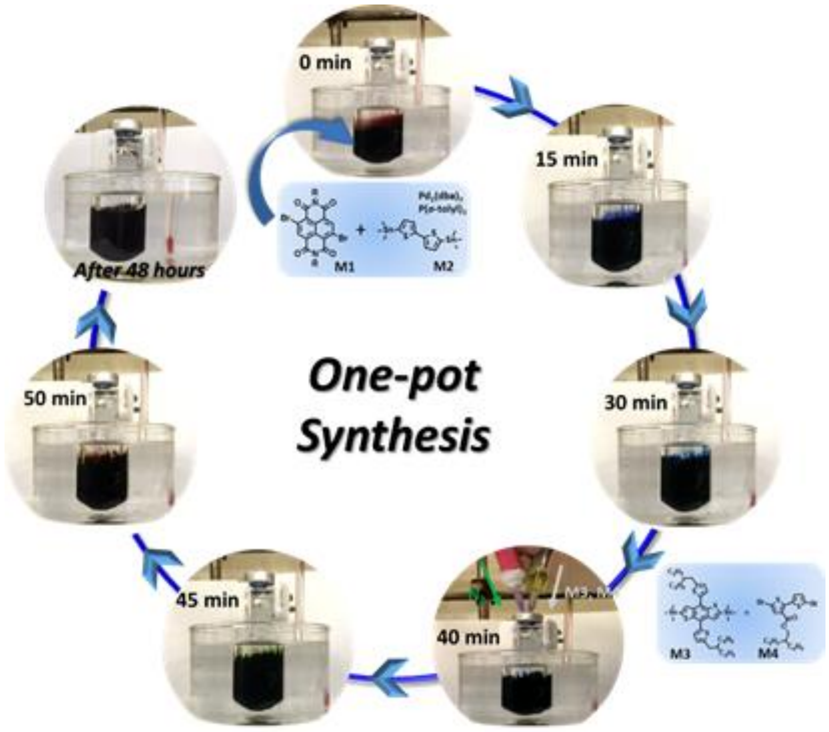

Figure S1. Photographs showing the progress of one-pot synthesis.

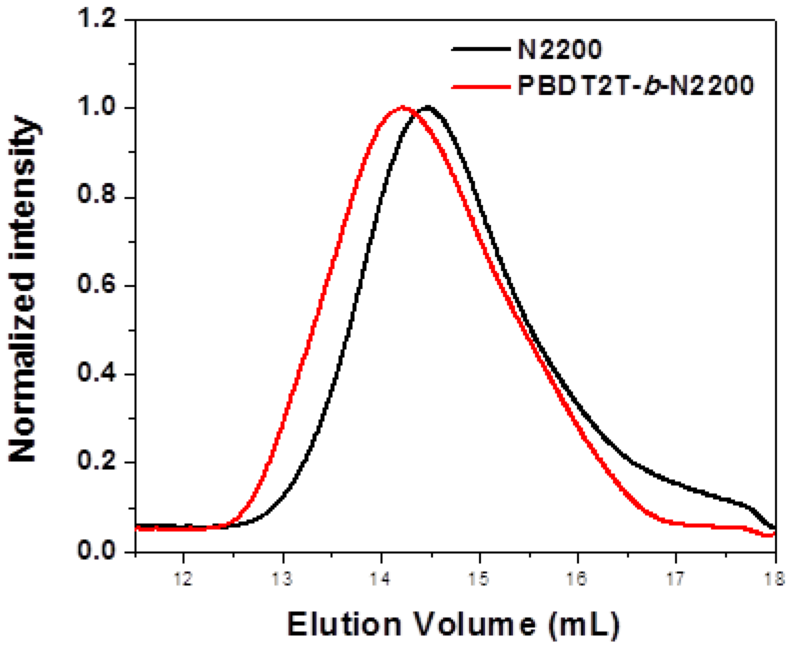

**Polymer**	M_n **(kg mol^{-1})**	**PDI**
**N2200 (40 min)**	20.9	3.28
**PBDT2T-*b*-N2200**	33.3	3.21

Figure S2. Number average molecular weight (M_n) and polydispersity index (PDI) data obtained from gel permeation chromatography.

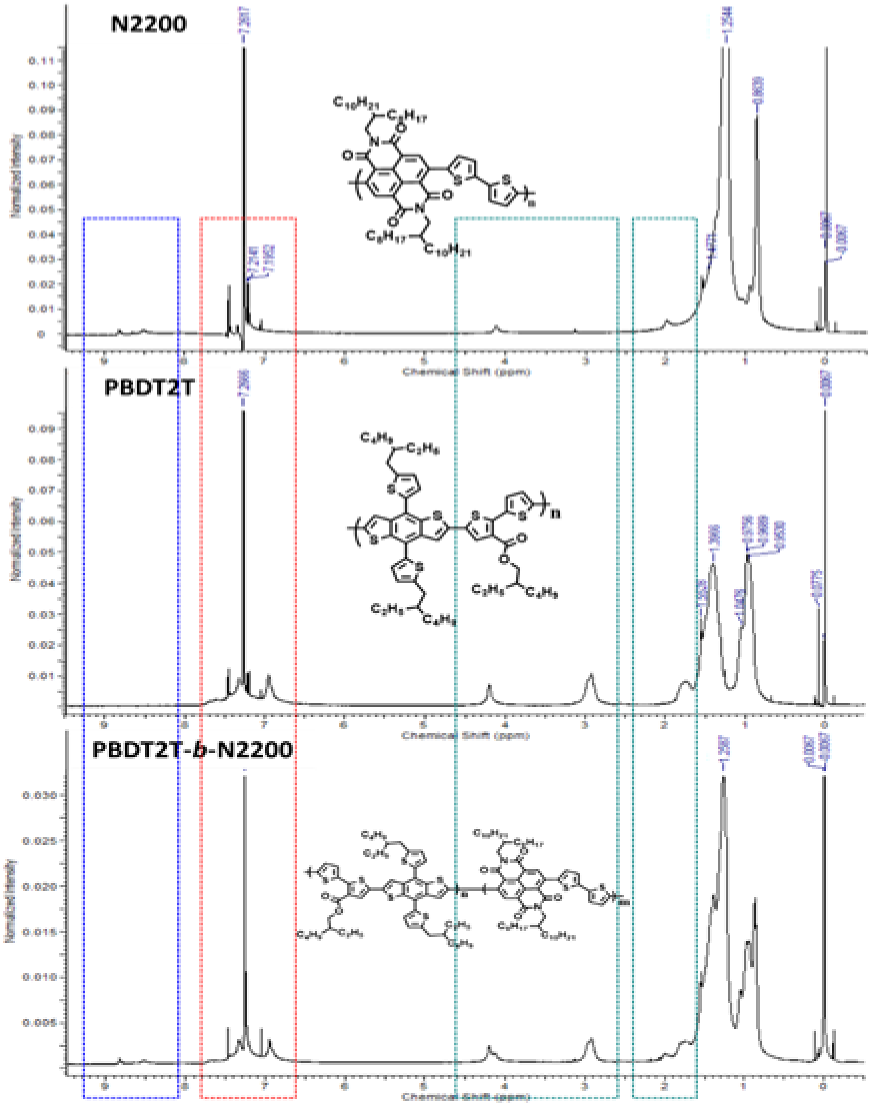

Figure S3. ^{1}H NMR spectra of PBDT2T, N2200, and PBDT2T-*b*-N2200.

Table S1. Elemental analysis data.

Sample	Analysis	Unit	Method	LOD	Result
PBDT2T-*b*-N2200	Nitrogen	%	EA	0.05	1.46
	Carbon	%	EA	0.05	71.33
	Hydrogen	%	EA	0.05	7.83
	Sulfur	%	EA	0.05	11.82

*LOD: limit of detection

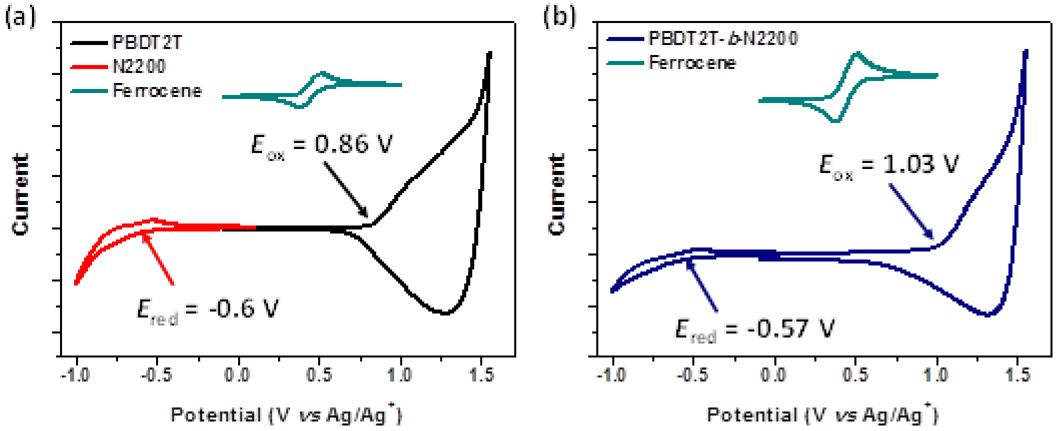

Figure S4. Cyclic voltammograms of (a) PBDT2T, N2200 and (b) PBDT2T-*b*-N2200. *Sample: film on Pt electrode.

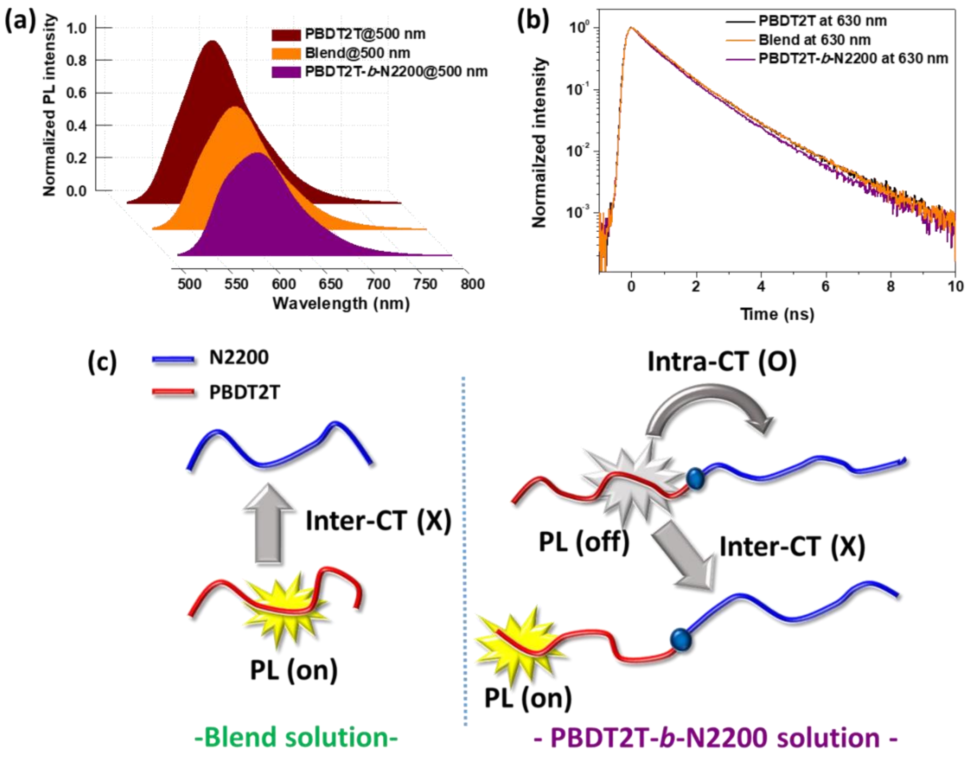

Figure S5. (a) steady state PL spectra measured in chloroform. (b) transient PL signals of PBDT2T, blend, and PBDT2T-*b*-N2200 films in chloroform (λ_{em} = 630 nm) after excitation at λ_{ex} = 500 nm. The concentration of PBDT2T, blend, and PBDT2T-*b*-N2200 films in chloroform is ~10^{-5} M. (c) Schematic representation of photoinduced CT between a donor and acceptor units in dilute solutions.

Table S2. Transient PL fit parameters of the PBDT2T, blend of PBDT2T and N2200, and PBDT2T-*b*-N2200 in chloroform (~10^{-5} M)

Polymer	A_1	τ_1(ns)	A_2	τ_2(ns)	A_3	τ_3(ns)	τ_{avg}(ns)
**PBDT2T**	0.73	0.80	0.27	1.6	-	-	1.0
**Blend**	0.66	0.76	0.34	1.5	-	-	1.0
**PBDT2T-*b*-N2200**	0.15	0.18	0.70	0.87	0.15	1.7	0.89

The average lifetime was calculated using $\tau_{avg} = \Sigma_i A_i \tau_i / \Sigma_i A_i$.

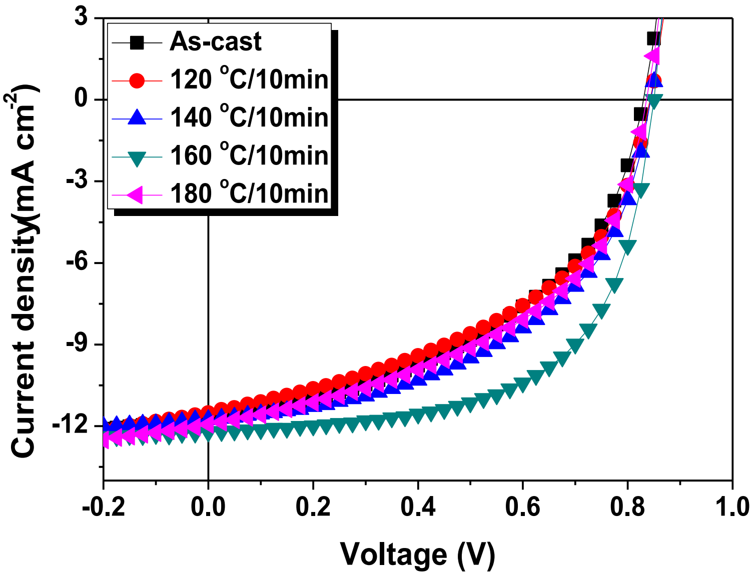

Figure S6. The *J–V* curves of the inverted devices of PBDT2T-*b*-N2200 processed from various annealing temperatures under simulated illumination of AM 1.5G at 100 mW cm^{-2}

Table S3. Summary of the photovoltaic properties of the devices processed under various annealing temperatures (AM 1.5G illumination 100 mW cm^{-2}).

Active layer	Annealing condition	V_{oc} (V)	J_{sc} (mA cm^{-2})	FF	PCE (%)	R_s (Ω cm^2)	R_{sh} (kΩ cm^2)
PBDT2T-*b*-N2200	As-cast	0.83	11.63	0.47	4.54	8.98	0.36
	120 °C/10 min	0.84	11.46	0.47	4.52	10.98	0.28
	140 °C/10 min	0.84	11.70	0.51	5.01	9.63	0.52
	160 °C/10 min	0.85	12.21	0.62	6.43	7.63	0.95
	180 °C/10 min	0.84	11.90	0.49	4.89	8.97	0.30

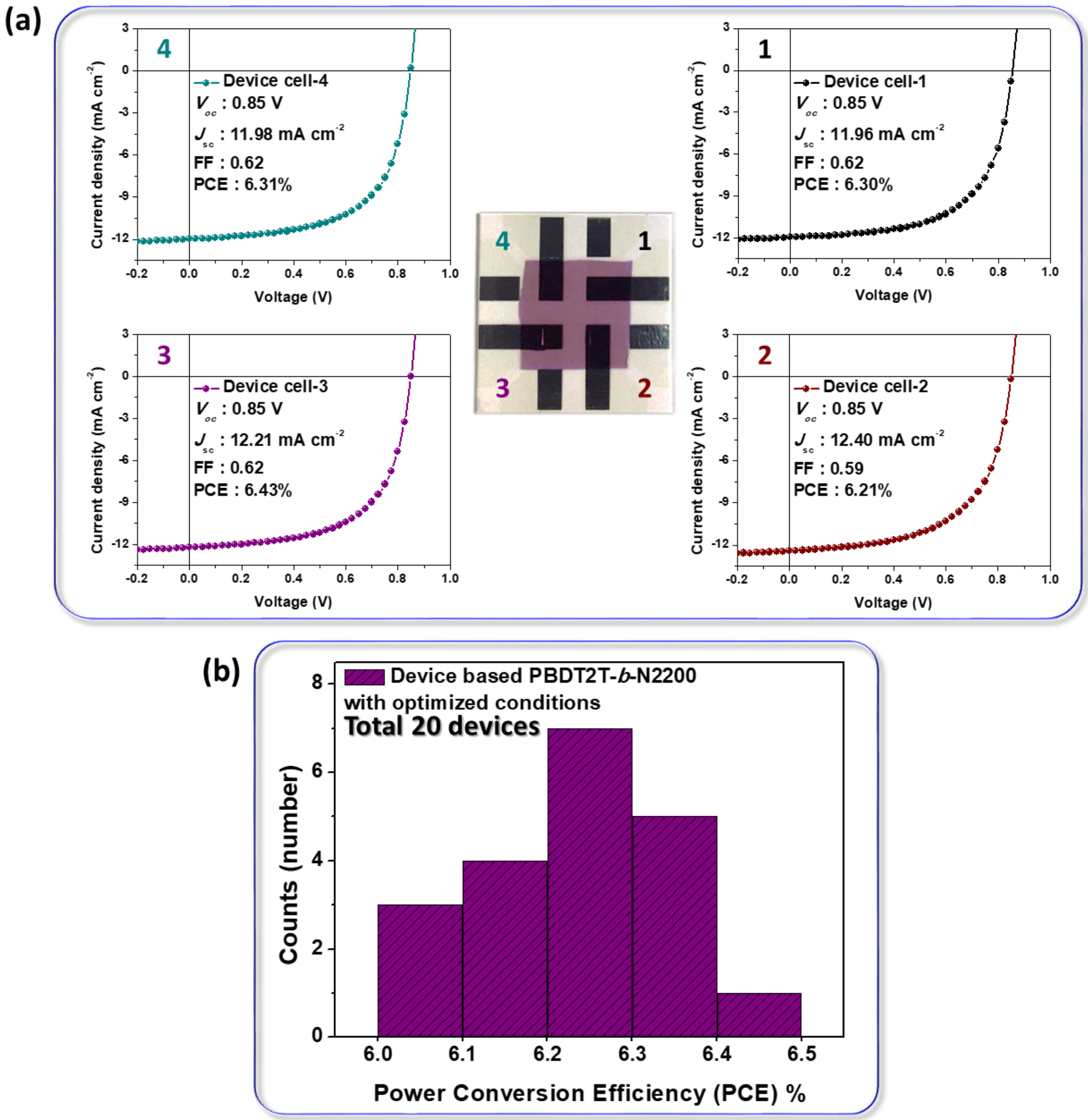

Figure S7. (a) Reproducibility of the *J–V* curves from different PSCs. The active layer is an annealed film of PBDT2T-*b*-N2200. (b) PCE distribution of optimized PSC devices (e.g. active layer annealed at 160 °C for 10 min.). The histogram shows the performance distribution of the 20 devices with the highest efficiency measured in this study.

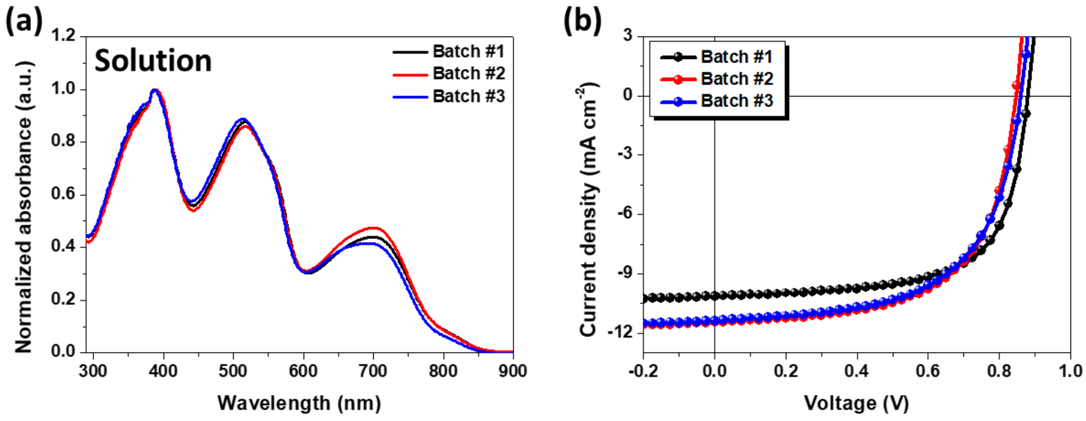

Figure S8. Reproducibility of (a) normalized UV-vis absorption spectra and (b) the *J*–*V* curves from different batches #1, #2, and #3 PSCs. The active layer was an annealed film of PBDT2T-*b*-N2200.

Table S4. Summary of the photovoltaic properties of the devices processed under batch #1, #2, and #3. (AM 1.5G illumination 100 mW cm^{-2}).

	Absorption band (nm)	Annealing condition	V_{oc} (V)	J_{sc} (mA cm^{-2})	FF	PCE (%)
**Batch #1**	387, 517, 702	160 °C/10 min	0.88	10.13	0.67	5.97
**Batch #2**	388, 517, 700		0.85	11.43	0.62	6.02
**Batch #3**	388, 514, 695		0.86	11.38	0.61	5.96

To confirm the reproducibility of the characteristics of the block copolymers and their corresponding PSC devices, polymer syntheses have been carried out three times under the same conditions (Batches #1, #2, and #3). According to **Figure S8(a)**, the content of AB seems to be different in the three polymers. As a result, Batch #2 demonstrated better device characteristics; thus, we performed fundamental property evaluation and optimization of the corresponding device performance using the polymer obtained in Batch #2.

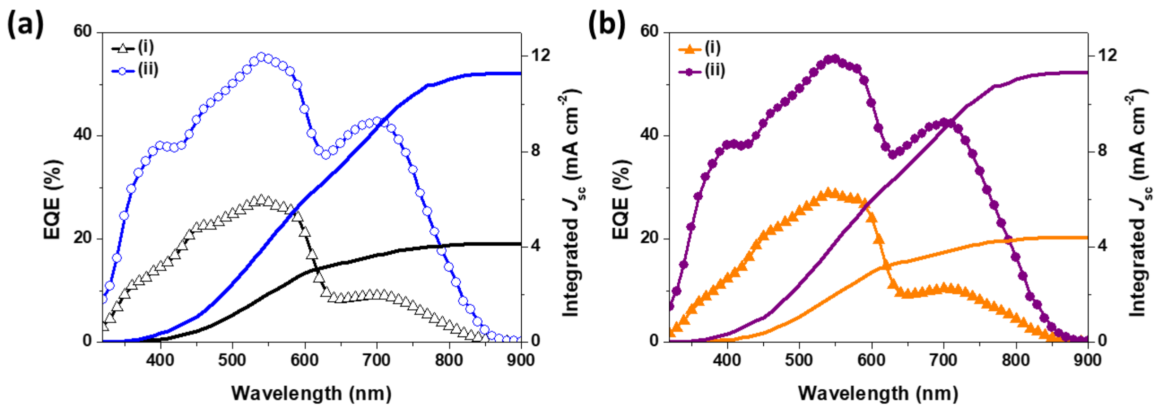

Figure S9. EQE spectra and integrated current densities. (a) PSCs with as-cast films. (i) as-cast blend film, (ii) as-cast PBDT2T-*b*-N2200 film, (b) PSCs with annealed films. (i) annealed blend film, (ii) annealed PBDT2T-*b*-N2200 film.

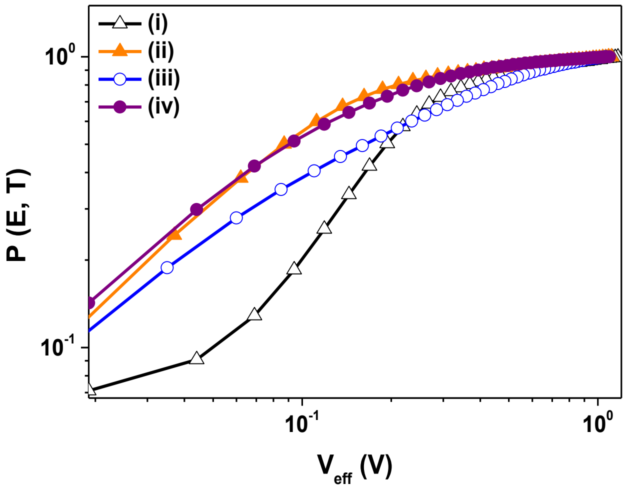

Figure S10. Plots of J_{ph}/J_{sat} versus effective voltage (V_{eff}). (i) as-cast blend film, (ii) annealed blend film, (iii) as-cast PBDT2T-*b*-N2200 film, and (iv) annealed PBDT2T-*b*-N2200 film.

Table S5. Maximum exciton generation rate (G_{max})

Active layer	J_{sat}	q	Thickness (L)	G_{max}
**Blend**	47.31 A m^{-2}	1.602 × 10^{-19} A·s	95 × 10^{-9} m	3.10 × 10^{27} m^{-3} s^{-1}
**PBDT2T-*b*-N2200**	123.83 A m^{-2}	1.602 × 10^{-19} A·s	93 × 10^{-9} m	8.31 × 10^{27} m^{-3} s^{-1}

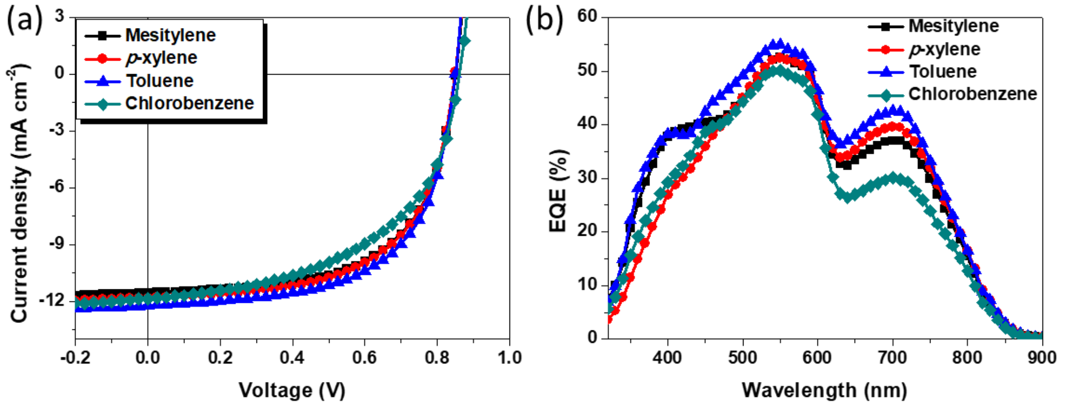

Figure S11. (a) *J–V* characteristics and (b) EQE spectra of PSCs with active layers of PBDT2T-*b*-N2200. The active layers were made from mesitylene, *p*-xylene, toluene, and chlorobenzene solutions.

Table S6. Photovoltaic performance of PSCs with PBDT2T-*b*-N2200 under AM 1.5G illumination (100 mW cm^{-2}).

Solvent	Annealing condition	V_{oc} (V)	J_{sc} (mA cm^{-2})	FF	PCE (%)	R_s (Ω cm^2)	R_{sh} (kΩ cm^2)
Mesitylene	160 °C /10 min	0.85	11.53	0.62	6.07	5.70	1.14
p-xylene		0.85	11.79	0.61	6.11	8.06	1.08
Toluene		0.85	12.21	0.62	6.43	7.63	0.95
Chlorobenzene		0.86	11.81	0.53	5.38	7.97	0.55

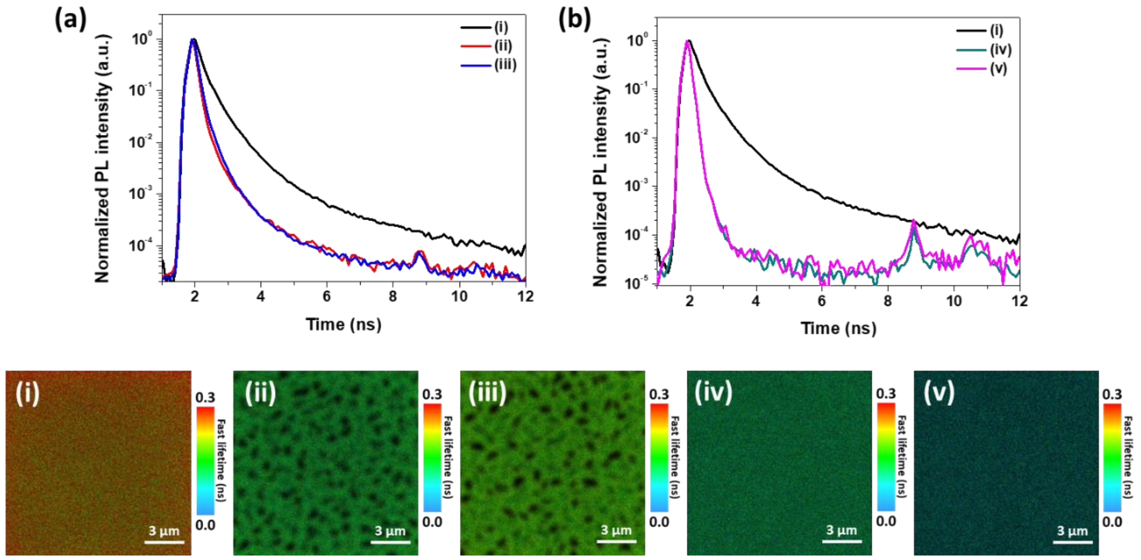

Figure S12. Time-resolved photoluminescence (TRPL) of the PBDT2T film: (a) PBDT2T:N2200 blend film and (b) PBDT2T-*b*-N2200 film (detected at 600–700 nm). (i-v) The TRPL lifetime images of the PBDT2T, blend, and PBDT2T-*b*-N2200 films (dark spots denote areas of very low photon intensity). (i) As-cast PBDT2T film, (ii) as-cast blend film, (iii) annealed blend film, (iv) as-cast PBDT2T-*b*-N2200 film, and (v) annealed PBDT2T-*b*-N2200 film.

Table S7. TRPL fit parameters of the neat PBDT2T, blend, and PBDT2T-*b*-N2200 films.

Polymer	A_1	τ_1 (ns)	A_2	τ_2 (ns)	A_3	τ_3 (ns)	τ_{avg} (ns)	Quenching efficiency [c]
**PBDT2T**	2315.829	0.187	420.828	0.515	5.750	2.230	0.242	-
**Blend** [a]	2175.444	0.098	58.232	0.355	1.536	1.341	0.106	0.56
**Blend** [b]	3263.880	0.108	155.294	0.325	2.129	1.354	0.119	0.51
**PBDT2T-*b*-N2200** [a]	1278.893	0.072	2.353	0.341	-	-	0.072	0.70
**PBDT2T-*b*-N2200** [b]	754.304	0.071	1.620	0.323	-	-	0.071	0.71

[a] As-cast [b] thermal annealing at 160 °C /10 min, [c] quenching efficiencies were calculated using the relationship 1-($\tau_{avg}/\tau_{avg,PBDT2T}$), where τ_{avg} is the average exciton lifetime, $\tau_{avg,PBDT2T}$ is the average exciton lifetime of the neat PBDT2T film. The average lifetime was calculated using $\tau_{avg} = \Sigma_i A_i \tau_i / \Sigma_i A_i$.

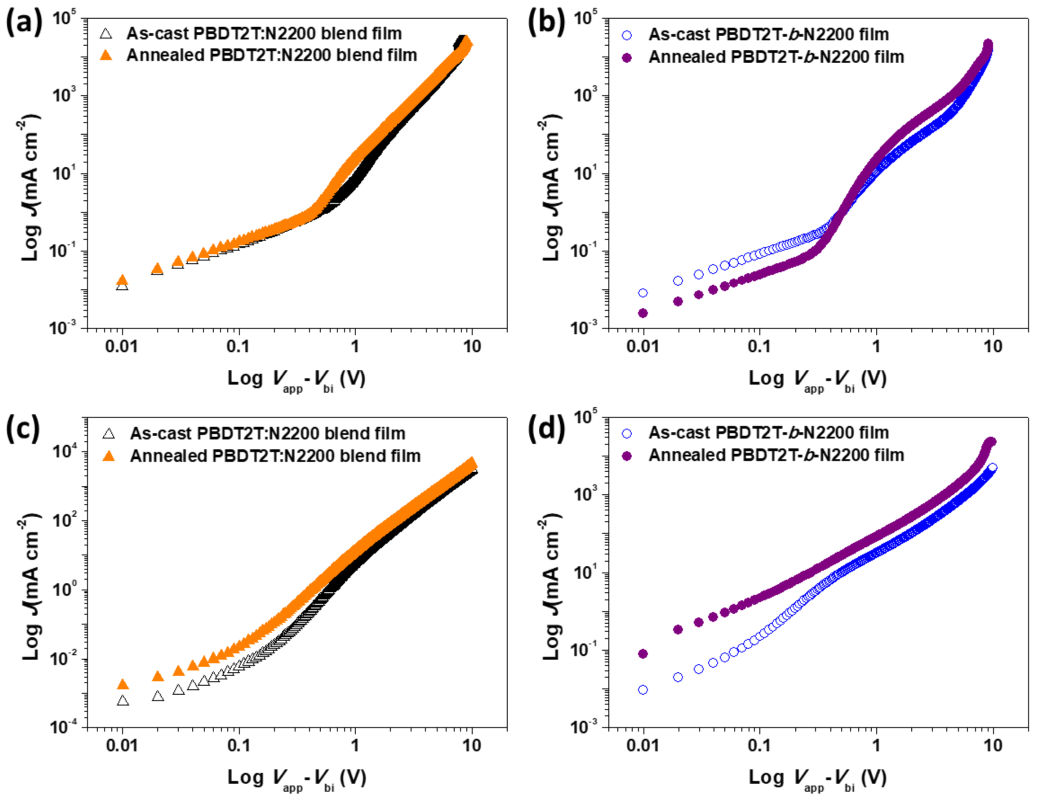

Figure S13. Space-charge-limited current *J*–*V* characteristics of the PBDT2T:N2200 blend film and the PBDT2T-*b*-N2200 films under dark conditions for (a), (b) hole-only devices and (c), (d) electron-only devices.

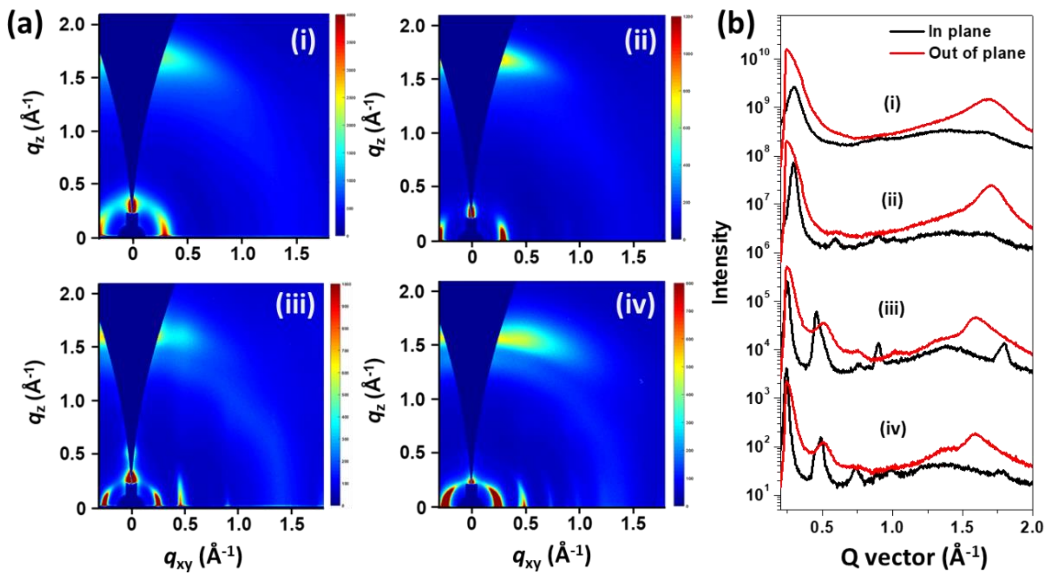

Figure S14. Grazing incidence wide-angle X-ray diffraction for PBDT2T and N2200 films: a) 2D diffraction pattern and b) in-plane (black lines) and out-of-plane (red lines) profiles. (i) As-cast PBDT2T film, (ii) annealed PBDT2T film, (iii) as-cast N2200 film, and (iv) annealed N2200 film.

Table S8. Structural parameters of PBDT2T and N2200 in thin films obtained from GIWAXD measurements.

Polymer	Annealing condition.	Plane	Direction	q (Å^{-1})	d-spacing (Å)	FWHM (Å)	Coherence length (Å)
PBDT2T	As-cast	Out	(010)	1.68	3.73	0.20	31.28
		In	(100)	0.29	21.34	0.08	72.20
	160 °C /10 min	Out	(010)	1.69	3.70	0.14	43.21
		Out	(200)	-	-	-	-
		In	(100)	0.29	21.34	0.06	102.26
		In	(200)	0.59	10.65		
		In	(300)	0.89	7.00		

Polymer	Annealing condition.	Plane	Direction	q (Å^{-1})	d-spacing (Å)	FWHM (Å)	Coherence length (Å)
N2200	As-cast	Out	(010)	1.60	3.92	0.12	45.81
		Out	(200)	0.51	12.23	-	-
		Out	(300)	0.75	8.31	-	-
		In	(100)	0.24	25.81	0.02	227.45
		In	(001)	0.45	13.71	-	-
		In	(200)	0.48	12.91	-	-
		In	(300)	0.74	8.38	-	-
		In	(002)	0.89	6.98	-	-
	160 °C /10 min	Out	(010)	1.60	3.92	0.12	46.20
		Out	(200)	0.51	12.23	-	-
		Out	(300)	0.75	8.31	-	-
		In	(100)	0.24	25.81	0.02	272.67
		In	(001)	0.45	13.78	-	-
		In	(200)	0.48	12.91	-	-
		In	(300)	0.73	8.55	-	-
		In	(002)	0.89	7.03	-	-

$L_{coherence} = 2\pi K/\mathrm{FWHM}$ $(K \approx 0.93)$

Table S9. lists the structure parameters of the PBDT2T-*b*-N2200 and PBDT2T/N2200 blend films obtained from the GIWAXD measurements.

Polymer	Annealing condition.	Plane	Direction	q (Å^{-1})	d-spacing (Å)	FWHM (Å)	Coherence length (Å)
PBDT2T-*b*-N2200	As-cast	Out	(010)	1.65	3.86	0.24	23.51
		Out	(200)	0.51	12.28	-	-
		In	(100)	0.25	24.43	0.05	102.13
		In	(001)	0.45	13.85	-	-
		In	(200)	0.48	-	-	-
	160 ^{o}C /10 min	Out	(010)	1.65	3.79	0.20	27.91
		Out	(200)	0.51	12.28	-	-
		In	(100)	0.25	24.43	0.04	123.30
		In	(001)	0.45	13.85	-	-
		In	(200)	0.48	-	-	-

Polymer	Annealing condition.	Plane	Direction	q (Å^{-1})	d-spacing (Å)	FWHM (Å)	Coherence length (Å)
Blend	As-cast	Out	(010)	1.65	3.87	0.33	17.58
		Out	(200)	0.50	12.55	-	-
		In	(100)	0.24<br>0.28	25.34<br>21.66	-	-
		In	(001)	0.45	13.78	-	-
		In	(200)	0.48	12.90	-	-
		In	(002)	0.90	6.93	-	-
	160 ^{o}C /10 min	Out	(010)	1.68	3.72	0.25	22.64
		Out	(200)	0.50	12.49	-	-
		Out	(300)	0.71	8.82	-	-
		In	(100)	0.24<br>0.28	26.04<br>21.66	-	-
		In	(001)	0.45	13.85	-	-
		In	(200)	0.48	13.08	-	-
		In	(300)	0.71	8.73	-	-
		In	(002)	0.89	7.00	-	-

$L_{coherence} = 2\pi K/\text{FWHM} \quad (K \approx 0.93)$

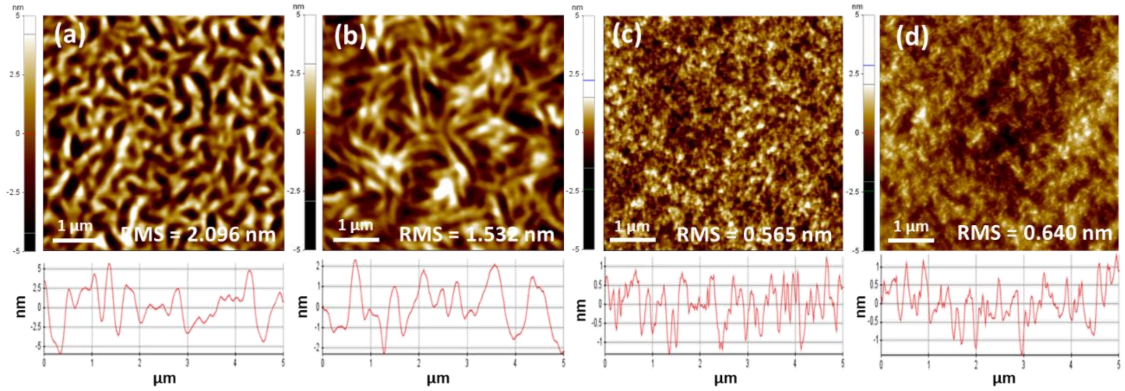

Figure S15. (a-d) AFM height images and surface profiles of thin films fabricated from a toluene solution with thermal annealing at 160 °C for 10 min. (a, c) before aging and (b, d) after aging under ambient conditions in the dark for up to 1,020 h. (a, b) PBDT2T:N2200 blend film and (c, d) PBDT2T-*b*-N2200 film.

Additional References

S1. F. Pierini, M. Lanzi, P. Nakielski, S. Pawłowska, O. Urbanek, K. Zembrzycki, T. A. Kowalewski, *Macromolecules* **2017**, *50*, 4972.

S2. T. L. Nguyen, T. H. Lee, B. Gautam, S. Y. Park, K. Gundogdu, J. Y. Kim, H. Y. Woo, *Adv. Funct. Mater.* **2017**, *27*, 1702474.

S3. G. Feng, J. Li, Y. He, W. Zheng, J. Wang, C. Li, Z. Tang, A. Osvet, N. Li, C. J. Brabec, Y. Yi, H. Yan, W. Li, *Joule.* **2019**, *3*, 1765.
